# Beta-Pix-dynamin 2 complex promotes colorectal cancer progression by facilitating membrane dynamics

**DOI:** 10.1007/s13402-021-00637-6

**Published:** 2021-09-28

**Authors:** Seula Keum, Soo Jung Yang, Esther Park, TaeIn Kang, Jee-Hye Choi, Jangho Jeong, Ye Eun Hwang, Jung-Woong Kim, Dongeun Park, Sangmyung Rhee

**Affiliations:** 1 Department of Life Science, Chung-Ang University, Seoul, 06974 Republic of Korea; 2 Translational Research Program, Benaroya Research Institute at Virginia Mason, Seattle, WA 98101 USA; 3 School of Life Sciences, Seoul National University, Seoul, 08826 Republic of Korea

**Keywords:** Colorectal cancer, Metastasis, Beta-Pix, Dynamin 2, Cytoskeleton rearrangement, Src kinase

## Abstract

**Purpose:**

Spatiotemporal regulation of cell membrane dynamics is a major process that promotes cancer cell invasion by acting as a driving force for cell migration. Beta-Pix (βPix), a guanine nucleotide exchange factor for Rac1, has been reported to be involved in actin-mediated cellular processes, such as cell migration, by interacting with various proteins. As yet, however, the molecular mechanisms underlying βPix-mediated cancer cell invasion remain unclear.

**Methods:**

The clinical significance of βPix was analyzed in patients with colorectal cancer (CRC) using public clinical databases. Pull-down and immunoprecipitation assays were employed to identify novel binding partners for βPix. Additionally, various cell biological assays including immunocytochemistry and time-lapse video microscopy were performed to assess the effects of βPix on CRC progression. A βPix-SH3 antibody delivery system was used to determine the effects of the βPix-Dyn2 complex in CRC cells.

**Results:**

We found that the Src homology 3 (SH3) domain of βPix interacts with the proline-rich domain of Dynamin 2 (Dyn2), a large GTPase. The βPix-Dyn2 interaction promoted lamellipodia formation, along with plasma membrane localization of membrane-type 1 matrix metalloproteinase (MT1-MMP). Furthermore, we found that Src kinase-mediated phosphorylation of the tyrosine residue at position 442 of βPix enhanced βPix-Dyn2 complex formation. Disruption of the βPix-Dyn2 complex by βPix-SH3 antibodies targeting intracellular βPix inhibited CRC cell invasion.

**Conclusions:**

Our data indicate that spatiotemporal regulation of the Src-βPix-Dyn2 axis is crucial for CRC cell invasion by promoting membrane dynamics and MT1-MMP recruitment into the leading edge. The development of inhibitors that disrupt the βPix-Dyn2 complex may be a useful therapeutic strategy for CRC.

**Supplementary Information:**

The online version contains supplementary material available at 10.1007/s13402-021-00637-6.

## Introduction

Globally, colorectal cancer (CRC) is the third most common cancer (10.2 % of all cancer cases) and the second most common cause of cancer-related mortality (9.2 %) [1]. The high CRC-related mortality rate is closely related to the occurrence of metastasis, which is characterized by the dissemination of aggressive cancer cells from the primary tumor to distant organs. Cell migration and invasion are aberrantly activated in cancer cells during metastasis. To invade surrounding tissues cancer cells, to a considerable extent, undergo cytoskeleton rearrangements with the dynamic assembly and disassembly of F-actin-based structures, such as membrane ruffles, blebs, filopodia, lamellipodia and invadopodia [2, 3].

Membrane protrusions are spatiotemporally regulated by classical Rho family small guanosine triphosphatases (GTPases), including RhoA, Rac1 and Cdc42, along with their downstream effectors [4–6]. The activity of classical Rho GTPases is controlled via reversible actions between Rho-specific guanine nucleotide exchange factors (GEFs) and GTPase-activating proteins by exchanging bound GDP nucleotides for GTP nucleotides and *vice versa*. Specifically, several GEFs are vital for promoting Rho GTPase-mediated cell migration. The Dbl and DOCK families of proteins have been identified as specific types of GEFs. Among them, beta-Pix (βPix), which activates Rac1 and Cdc42, is one of the most frequently investigated GEFs, particularly in terms of cell spreading and migration [7–9]. Previous studies have revealed that βPix promotes collective migration of endoderm cells and neural tube formation by activating downstream signaling including Rac1 and p21-activated kinase 2a (Pak2a) during embryonic development [9–11]. Mechanistically, βPix plays an active role in cell migration by forming several complexes. To date, the most notable βPix-mediated multi-protein complex is the βPix-GIT-PAK complex, known to induce Rac1-mediated actin reorganization at the membrane edge and to recycle focal adhesion (FA) components in an integrin-dependent manner [12, 13]. In addition, it has been found that the P-cadherin-βPix complex is required for Cdc42-induced cell polarity and mechanical forces during collective migration [14]. Therefore, βPix may exert certain intracellular functions related to its binding partner.

Overexpression of βPix has been observed in patients with breast cancer and CRC, indicating its potential as a cancer biomarker [15, 16]. Specifically, in CRC cells βPix has been found to enhance the transcriptional activity of β-catenin via direct binding, leading to cell proliferation regardless of GEF activity [17]. Considering the intracellular functions of βPix, it is reasonable to speculate that βPix may play a role in CRC cell motility or invasion. As yet, however, the specific binding protein that controls the function of βPix in CRC remains unknown.

Dynamin 2 (Dyn2), a large GTPase, participates in the endocytic pathway. To date, three dynamin isoforms (Dyn1, Dyn2 and Dyn3) have been identified. Dyn2 is ubiquitously expressed, whereas Dyn1 is expressed only in neuronal cells and Dyn3 in the brain, lung and testis [18]. It has been reported that Dyn2 is necessary for embryonic development and is involved in regulating integrin endocytosis and actin filament distribution during muscle maturation [19–21]. In addition to the scission of newly formed vesicles from the plasma membrane, Dyn2 is reportedly responsible for cell migration by controlling microtubule and actin cytoskeleton dynamics. Furthermore, Dyn2 has been found to be involved in metastatic activity by regulating microtubule-dependent FA dynamics [22], and the effect of Dyn2 on cell motility has been found to be largely associated with its ability to control F-actin rearrangement, including the assembly in lamellipodia and the promotion of membrane ruffling [23]. Moreover, recent studies have shown that the interaction of Dyn2 with α-actinin promotes invadopodia formation, resulting in pancreatic cancer cell invasion. This finding suggests that Dyn2 can act as a scaffold protein to spatiotemporally modulate the actin cytoskeleton and its regulatory proteins for cancer metastasis [24–26].

In the present study, we show that βPix interacts with the proline-rich domain (PRD) of Dyn2 via the SH3 domain of βPix, and co-localizes at the membrane edge. The βPix-Dyn2 complex, promoted by the Src kinase-induced phosphorylation of tyrosine at position 442 in βPix, is crucial for Rac1-mediated membrane ruffling and CRC cell invasion. Interestingly, blockade of βPix-Dyn2 complex formation by intracellular delivery of an anti-βPix-SH3 antibody impaired cell invasion. These results suggest that disruption of the βPix-Dyn2 complex may be a therapeutic strategy for treating CRC.

## Materials and methods

### Clinical data mining

Expression profiles of *ARHGEF7*, which encodes βPix, in patients with CRC were obtained from the Oncomine (www.oncomine.org) and Gene Expression Omnibus (GEO) (www.ncbi.nlm.nih.gov/geo) databases. For transcriptional analysis in Oncomine, data with *p* < 0.0001, fold change > 2, and gene rank < 10 % were used. For GEO analysis, accession numbers GSE20916 and GSE32474 were selected. Analysis of βPix protein expression in patients with CRC was performed using the Human Protein Atlas (www.proteinatlas.org). Additional gene expression datasets for CRC (GSE29621 and GSE14333) were downloaded for overall and recurrence-free survival analyses. The distribution of βPix-Dyn2 expression in patients with CRC across the three lymph node stages was analyzed using The Cancer Genome Atlas (TCGA; Pan Cancer Atlas, 2018) from the cBioportal database.

### Antibodies and reagents

In the present study, we produced monoclonal anti-βPix-SH3 and polyclonal anti-βPix-GBD antibodies against purified GST-SH3 and GST-GBD proteins. In addition, we procured antibodies against Dyn2 (C-18, #sc-6400; Santa Cruz Biotechnology, Dallas, TX, USA), E-cadherin (24E10, #3195; Cell Signaling Technology, Danvers, MA, USA), MT1-MMP (L-15, #sc-12367; Santa Cruz Biotechnology), GAPDH (6C5, #sc-32233; Santa Cruz Biotechnology), Cortactin (H-191; #sc-11408; Santa Cruz Biotechnology), GST (B-14, #sc-138; Santa Cruz Biotechnology), c-Myc (9E10, #sc-40; Santa Cruz Biotechnology), FLAG (M2, #F1804; Sigma-Aldrich, St. Louis, MO, USA), GFP (B-2, #sc-9996; Santa Cruz Biotechnology), Rac1 (#610651; BD Transduction Laboratory, San Jose, CA, USA), and phosphotyrosine (4G10; #05-321; Millipore, Burlington, MA, USA). In addition, epidermal growth factor (EGF, E9644; Sigma-Aldrich), PP2, a Src kinase inhibitor (#529573; Calbiochem, La Jolla, CA, USA), poly L-lysine hydrobromide (#P6282; Sigma-Aldrich), fibronectin (#F2006; Sigma-Aldrich) and Matrigel (#354234; Corning, Corning, NY, USA) were used.

### Plasmids

Dyn2-GFP was provided by Mark A. McNiven (Mayo Clinic and Foundation, MN, USA). Paxillin cloned into pME18S-FL3 was purchased from the Korea Human Gene Bank (KHGB, #KU016281; Daejeon, South Korea). Expression vectors were generated by digesting pFlagCMV2 (#E7033; Sigma-Aldrich), pcDNA3.1 myc/His A (#V800-20; Invitrogen, Carlsbad, CA, USA), pEGFP-C1 (#6084-1; Clontech, Palo Alto, CA, USA) and pEGFP-N1 (#6085-1; Clontech) vectors with restriction enzymes. βPix mutants, i.e., SH3 (W43K), DH, Y442F, Y442E and Dyn2 (R834A and K44A), were generated using a QuickChange™ Site-Directed Mutagenesis Kit (#200518; Agilent Technologies, Santa Clara, CA, USA) following the manufacturer’s instructions. The following lentiviral shRNA oligonucleotides were used: human *βPix* shRNA #1 (5′-GCAAATGCTCGTACAGTCT-3′) and shRNA #2 (5′-CGACAGGAATGACAATCAC-3′) targeting the coding region of *βPix*, human *βPix* shRNA #3 (5′-TGCGAATGGAGACGATCAAAC-3′) targeting the 3′ untranslated region (UTR) of *βPix*, human *Dyn2* shRNA #1 (5′-ATGTAGGGCAGGCCTTCTATA-3′) targeting the 3′UTR of *Dyn2*, and shRNA #2 (5′-CCCGTTGAGAAGAGGCTACAT-3′) targeting the coding region of *Dyn2*. These shRNA oligos were cloned into a pLKO.1 vector (#10878; Addgene, Cambridge, MA, USA). For overexpressing Flag-*βPix* using the lentiviral system, the pLenti-G418 vector generated from pLenti-puro (#39481; Addgene) was used. All constructs were verified using DNA sequencing.

### Mammalian cell culture and transfection

The human colorectal adenocarcinoma LoVo, SW480 and DLD-1 cell lines were gifted by Eok-Soo Oh (Ewha Womans University). LoVo cells were maintained in Roswell Park Memorial Institute 1640 medium (RPMI-1640; #31800022; Gibco, Grand Island, NY, USA), SW480 cells were maintained in Dulbecco’s modified Eagle’s medium: Nutrient Mixture F-12 (DMEM/F-12; #12500062; Gibco) and DLD-1 cells were maintained in DMEM (#12100046; Gibco) supplemented with 10 % heat-inactivated fetal bovine serum (FBS; #US-FBS-500; GW Vitek, Seoul, South Korea), 100 units/ml penicillin and 100 µg/ml streptomycin (#LS202-02; WelGENE, Daegu, South Korea). In addition, HEK293T cells were maintained in DMEM supplemented with 10 % FBS (#US-FBS-500; GW Vitek), 100 units/ml penicillin and 100 ug/ml streptomycin (#LS202-02; WelGENE). The cells were incubated at 37 °C in a humidified incubator with 5 % CO_2_. For transient transfection, 1–3 µg of plasmids was transfected into HEK293T cells using the calcium phosphate precipitation method. SW480 cells were transfected using Lipofectamine 3000 Reagent (#L3000015; Invitrogen) according to the manufacturer’s instructions.

### Generation of stable cell lines using a lentiviral system

*βPix* and *Dyn2* knockdown SW480 cell lines were generated using a lentiviral system. shRNA constructs were packaged with helper plasmids pMD2.G and psPAX2 (#12259 and #12260; Addgene), which were co-transfected into HEK293T cells. Lentiviral particles containing shRNA constructs were harvested from HEK293T cells after 72 h and infected into SW480 cells using 8 µg/ml polybrene. For establishing stable knockdown cell lines, cell selection was performed by treatment with 1 µg/ml puromycin (#P8833; Sigma-Aldrich). Depleted expression of βPix and Dyn2 was verified using Western blotting. The absence of off-target shRNA effects was verified using quantitative reverse transcription-polymerase chain reaction (RT-qPCR; Supplementary Table S1). Overexpression of Flag-*βPix* in LoVo cells was also performed using a lentiviral system, and the cells were selected using 500 µg/ml OmniPur® G418 Sulfate (#5.09290; Calbiochem). Overexpression of Flag-*βPix* was verified using Western blotting.

### RT-qPCR

For isolating total RNA, SW480 cells were lysed using RNAiso Plus reagent (#9109; TaKaRa, Tokyo, Japan) according to the manufacturer’s instructions. In brief, 1 µg RNA was used to synthesize complementary DNA using PrimeScript™ reverse transcriptase (#2680; TaKaRa). qPCR was performed using SYBR Premix Ex Taq II (#RR820; TaKaRa) and QuantStudio 3 (Applied Biosystems, Foster City, CA, USA). Gene expression levels were calculated using the 2^−ΔΔCt^ method and normalized to the Ct value of *GAPDH*. The primers used for qPCR are shown in Supplementary Table S2.

### GST pull-down assay

GST and GST-SH3 proteins were expressed in *Escherichia coli* BL21 and purified using Glutathione Sepharose 4B (#17-0756-01; GE Healthcare, Buffalo Grove, IL, USA), as described previously [27, 28]. HEK293T cells were washed with ice-cold phosphate-buffered saline (PBS) and lysed with pull-down buffer (50 mM Tris-Cl, pH 7.4, 100 mM NaCl, 0.5 % Triton X-100, 5 % glycerol, 5 mM MgCl_2_, 1 mM DTT, 20 mM NaF, 1 mM aprotinin, 1 mM leupeptin, and 1 mM pepstatin). After centrifugation at 21,000 × *g* for 15 min at 4 °C, the supernatant was incubated with 5 µg GST or GST-SH3 protein in pull-down buffer for 1 h at 4 °C and combined with 20 µl Glutathione Sepharose 4B. After incubation, the beads were washed three times with pull-down buffer, after which Western blotting was performed. For the GST-PBD pull-down assay, GST-PBD purified protein, a p21-binding domain of PAK1, was employed. HEK293T cells transfected with the indicated vectors were lysed with pull-down buffer and incubated with 5 µg GST-PBD protein for 1 h at 4 °C, followed by incubation with 20 µl Glutathione Sepharose 4B. Active Rac1 was pulled down and visualized using Western blotting with an anti-Rac1 antibody (#610651; BD Transduction Laboratory).

### Immunoprecipitation

At 24–36 h post-transfection, cells were washed with ice-cold PBS and lysed with IP buffer (50 mM HEPES, pH 7.4, 150 mM NaCl, 0.5 % NP 40, 1 mM EGTA, 1 mM EDTA, 20 mM NaF, 1 mM aprotinin, 1 mM leupeptin, and 1 mM pepstatin). Then, whole-cell lysates were centrifuged at 15,000 rpm for 15 min at 4 °C, and the concentration of the lysates was determined using the Bradford protein assay (#5000006; Bio-Rad, Hercules, CA, USA). In brief, 1 mg lysate was incubated with the appropriate antibodies for 2 h, followed by incubation with Protein A-Sepharose (#P3391, Millipore) for 1 h. The immunoprecipitates were washed with IP buffer and analyzed using Western blotting.

### Western blotting

SW480 cells were lysed with sodium dodecyl sulfate (SDS) lysis buffer (2 % SDS, 1 mM Na_3_VO_4_, 50 mM NaF, 1 mM DTT, and 1 mM PMSF). Then, PVDF membrane-transferred proteins were incubated with primary antibodies for 12–16 h at 4 °C. Horseradish peroxidase-conjugated secondary antibodies (Jackson ImmunoResearch Laboratories, West Grove, PA, USA) were added for 1–2 h at 24 °C. Protein signals were detected using enhanced chemiluminescence (ECL; #1705061; Bio-Rad) using the Fusion Solo S imaging system (VILBER, Collegien, France). Band densities of the proteins were measured using Evolution Capt software (VILBER).

### Immunocytochemistry

HEK293T cells were seeded on 0.1 mg/ml poly L-lysine- and 10 µg/ml fibronectin-coated coverslips and incubated in media for 15 min. SW480 cells, which were plated on Matrigel-coated coverslips, were fixed with 3.7 % paraformaldehyde for 15 min and permeabilized using 0.5 % Triton X-100 in PBS for 10 min. For blocking non-specific signals, the samples were incubated with blocking solutions: 2 % bovine serum albumin and 0.1 % Triton X-100 in PBS. The samples were stained with the indicated primary antibodies for 1 h at RT and then incubated with fluorescein-conjugated secondary antibodies for 1 h at RT after washing with 0.1 % Triton X-100 in PBS. Coverslips were mounted onto slides with Fluoromount-G (#0100-01; Southern Biotechnology Associates, Birmingham, AL, USA). The samples were evaluated using an ECLIPSE 80i fluorescence microscope (Nikon, Tokyo, Japan) and a Zeiss LSM700 confocal microscope (Zeiss, Oberkochen, Germany). Images were captured using a digital camera (DS-Qi2, Nikon) and processed using NIS-Elements image analysis software (Nikon). Pearson’s coefficients analysis to quantify protein colocalization was performed using NIS-Elements image analysis software (Nikon).

### 3-Dimensional (3D) sphere formation assay

SW480 cells (1 × 10^4^) were seeded in 6-well ultralow attachment plates (SPL 3D™ Cell Floater, #39706; SPL Life Sciences, Gyeonggi-do, South Korea) and incubated with media supplemented with 10 % FBS. Sphere formation was observed after seven days, and sphere diameters were measured using NIS-Elements image analysis software (Nikon). For immunocytochemistry, 3D spheres were fixed using 3.7 % paraformaldehyde for 15 min, and spheroids were added to adhesive microscope slides (HistoBond® microscope slides, #0810001; MARIENFELD, Lauda-Königshofen, Germany), followed by immunocytochemistry.

### Scratch wound healing migration and Matrigel invasion assays

For scratch wound healing assays, 2 × 10^5^ LoVo cells were seeded on 10 µg/ml Matrigel-coated 24-well culture plates with media containing 10 % FBS. After 16 h of incubation, the cells were scratched with a sterile 1-ml pipette tip and then incubated in media with 10 % FBS for 48 h. For the *in vitro* invasion assay, 2 × 10^5^ SW480 cells suspended in media without FBS were seeded on 10 µg/ml Matrigel-coated upper chamber membranes of a Transwell, with a pore size of 8 μm and a diameter of 6.5 mm (#35224; SPL Life Sciences). The inserts were supplemented with media containing 20 % FBS. LoVo cells (2 × 10^5^) suspended in media without FBS were seeded on the upper chamber membranes, supplemented with 20 % FBS. After 24 h of incubation, the cells at the bottom of the inserts were fixed with methanol and stained with 0.1 % crystal violet. The cells on the upper side of the Transwell membranes (non-invasive cells) were removed using a cotton swab and imaged randomly under 10× magnification using an Olympus CKX53 inverted microscope (Olympus, Tokyo, Japan), and the number of invasive cells was enumerated in each image.

Subsequently, we measured the activity of secreted matrix metalloproteinases (MMPs). To this end, 2 × 10^5^ SW480 cells were incubated for 16 h at 37 °C on 10 µg/ml Matrigel-coated 12-well culture plates with media containing 10 % FBS. After incubation, the cells were washed with serum-free media and incubated in fresh serum-free media for 24 h. Conditioned (supernatant) media were harvested and centrifuged at 200 × *g* for 3 min at 24 °C. Next, the supernatants were incubated with 10 µM fluorogenic MMP substrates (Mca-PLGL-Dpa-AR-NH2; #ES001; R&D Systems, Minneapolis, MN, USA) for 2 h at 37 °C. The fluorescence signals of samples were measured at 320-nm excitation and 405-nm emission using a Synergy HTX Multi-Mode Reader (BioTek, Winooski, VT, USA). The fluorescence signal subtracted from the background signal was normalized by the absorbance of crystal violet-stained cells at 550 nm, fixed using 3.7 % paraformaldehyde and incubated in 2 % SDS.

### Time-lapse imaging

To observe lamellipodia formation and FA dynamics, 7 × 10^4^ SW480 cells were seeded on Matrigel-coated glass-bottom plates 24 h post-transfection with plasmids. Next, the cells were serum-starved for 12 h and stimulated with 100 ng/ml EGF in a 5 % CO_2_ chamber at 37 °C. Images were captured under the indicated conditions using a Nikon ECLIPSE Ti2 inverted microscope system (Plan Apo λ 60X Oil) with a digital camera (DS-Qi2). To examine the rates of assembly and disassembly in individual FAs, each FA was tracked, and its intensity was measured over time, as described previously [29, 30]. The paxillin-GFP intensities in the time series were normalized using the intensity at the first point (each time point intensity/initial intensity; assembly rate calculations) or the last point (initial intensity/each time point intensity; disassembly rate calculations), and then converted by using the log scale. The log-transformed values were fitted to linear regression models to calculate assembly and disassembly rates. For the cell migration assay, 1 × 10^5^ SW480 cells were plated on Matrigel-coated plates and monitored at 30-min intervals for 12 h. Cell motility was analyzed using NIS-Elements image analysis software (Nikon). The mean square displacement was calculated according to the equation$: {(r\left(t\right)-r(0\left)\right)}^{2}$ = ${(x\left(t\right)-x(0\left)\right)}^{2}+{(y\left(t\right)-y\left(0\right))}^{2}$, where $r\left(t\right)$ is the position of a single cell at time $t$ and $r\left(0\right)$ is the initial position. Single-cell velocity was measured as $\sqrt{{(x\left(i\right)-x\left(i-1\right))}^{2}+{(y\left(i\right)-y\left(i-1\right))}^{2}} / dT$ at different time points.

### Preparation of gold nanoparticle (AuNP)-SH3 antibody complex

AuNPs (diameter: 15 nm) were functionalized with IgG aptamers (#NES002-01; NES, Seoul, South Korea) and conjugated with monoclonal anti-βPix-SH3 or normal mouse IgGs, as previously described [31]. AuNP-IgG and AuNP-SH3 conjugates were incubated with βPix-GFP- or Dyn2-GFP-expressing SW480 cells on Matrigel-coated glass. Antibody-conjugated AuNPs were visualized using fluorescein-conjugated secondary antibodies. The localization of βPix-GFP was evaluated after EGF stimulation for 20 min, followed by 4 h of serum starvation. For time-lapse video imaging, Dyn2-GFP-expressing SW480 cells treated with AuNP-SH3 were monitored for 30 min at 1-min intervals under EGF stimulation.

### Statistical analyses

Statistical analyses were performed using GraphPad Prism 8.0 (GraphPad Software, San Diego, CA, USA). One-way analysis of variance (one-way ANOVA) was performed to compare more than two groups. Student’s unpaired t-test was used to compare two groups. For all one-way ANOVA, Tukey’s multiple comparison test was employed as the post-hoc test. One-way ANOVA *F* values are displayed in each figure legend as F_(DFn, Dfd)_ (DFn as the df nominator and Dfd as the df denominator). Statistical significance was set at *p* < 0.05 (*p < 0.05, **p < 0.01, ***p < 0.001, ****p < 0.0001). Data are presented as the mean ± standard deviation (S.D.).

## Results

### Upregulation of βPix is highly associated with CRC progression

**Fig. 1:**
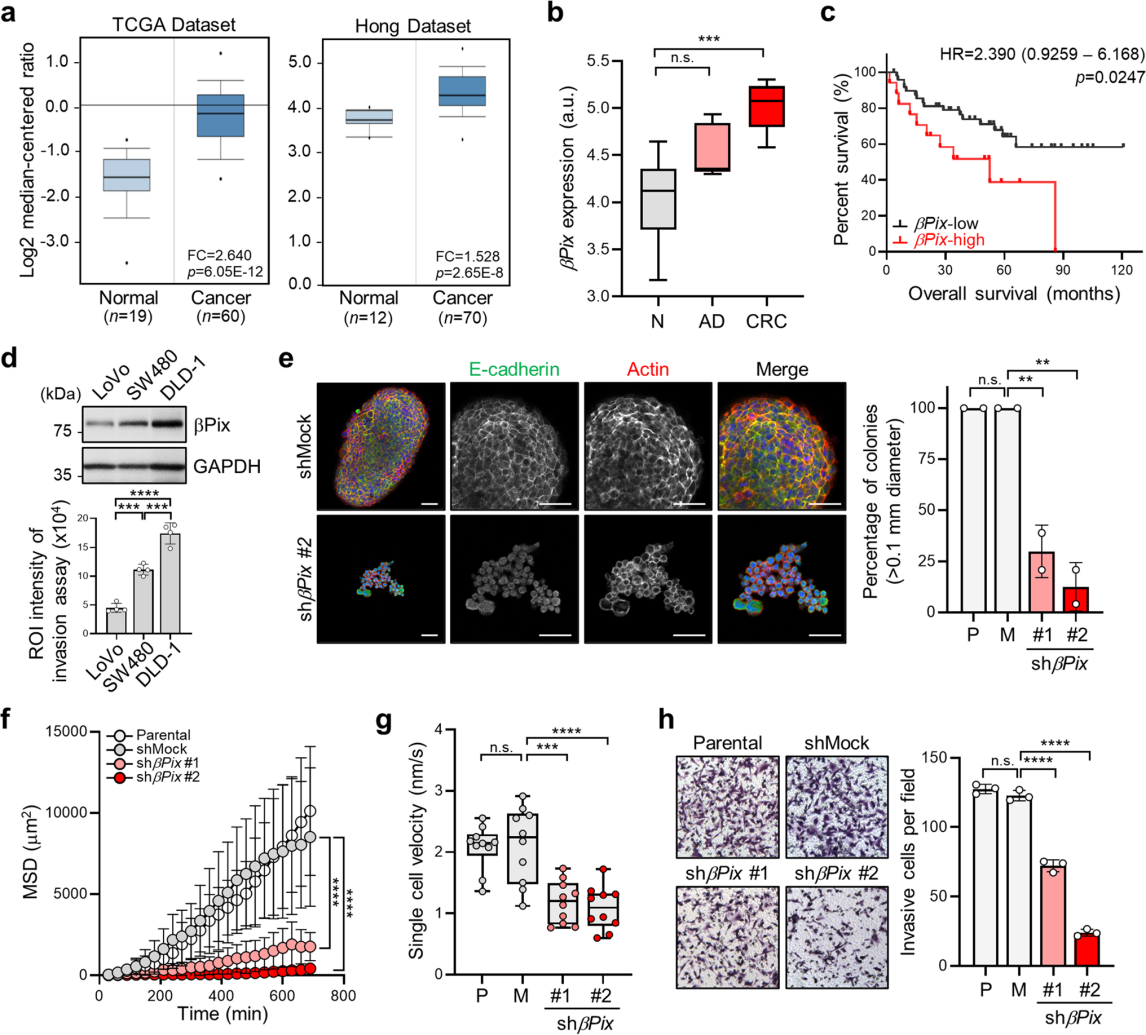
Increased βPix transcript levels are significantly related to poor prognosis in patients with CRC.** a** Box plots comparing the βPix levels between colorectal cancer (CRC) and normal tissues from Oncomine. **b** Analysis of βPix expression in CRC samples from GEO dataset GSE20916. N, normal and adjacent normal tissues; AD, adenoma tissues. Data are presented as box-and-whisker plots (min to max). Statistical analysis was performed using one-way ANOVA with Tukey’s multiple comparison test. One-way ANOVA, F_2, 17_ = 12.00. **c** Kaplan-Meier analysis of overall survival of 66 patients with CRC and high-βPix expression from GEO dataset GSE29621. **d** Western blots presenting βPix expression in various CRC cell lines with bar graphs comparing the invasiveness of cancer cell lines by measuring the region of interest (ROI) intensity of crystal violet in Transwell assays. Data are presented as the mean ± standard deviation (S.D.) from two independent experiments. Statistical analysis was performed using one-way ANOVA with Tukey’s multiple comparisons. One-way ANOVA, F_2,9_ = 105.2. **e** Representative images showing immunostaining of E-cadherin, actin and nuclei of tumor spheres from shMock and sh*βPix* #2 SW480 cells using fluorescein-conjugated antibodies. Scale bars, 50 μm. The bar graph on the right shows the percentage of colonies > 0.1 mm in diameter. Data are presented as the mean ± S.D. from two independent experiments and analyzed using one-way ANOVA, followed by Tukey’s multiple comparisons. One-way ANOVA, F_3,4_ = 55.87. **f** Mean squared displacement (MSD) of *βPix*-knockdown cells in single-cell tracking analysis by monitoring single cells with time-lapse video microscopy for 12 h at 30-min intervals under FBS conditions. MSDs are shown over 10 representative cell trajectories. One-way ANOVA, F_3, 36_ = 28.04. **g** Single-cell velocity of *βPix*-knockdown cells shown as box-and-whisker plots (min to max). One-way ANOVA, F_3, 36_ = 16.18. **h** Images of invasive cells in Transwell assays of *βPix-*knockdown cells under FBS conditions. Cell invasiveness was defined as the number of crystal violet-stained cells at the bottom of the lower chamber. Data are presented as the mean ± S.D. from three independent experiments. One-way ANOVA, F_3, 8_ = 565.2. **p < 0.01, ***p < 0.001, ****p < 0.0001, n.s. not significant

To determine the clinical significance of βPix in cancer progression, we analyzed its expression in various samples from patients with cancer using public databases. Accordingly, we found that βPix was specifically upregulated in CRC tissues when compared with normal tissues (Fig. 1a), whereas its expression in leukemia was controversial (*p* < 0.0001, fold change > 2, gene rank = top 10 %; Supplementary Fig. S1a). In addition, immunohistochemistry revealed overexpression of βPix in patients with CRC, verifying the results of the bioinformatic analysis (Supplementary Fig. S1b). GEO analysis revealed that the level of βPix expression correlated with the progression of CRC (Fig. 1b). In patients with CRC and elevated βPix expression, analysis of Kaplan–Meier plots revealed shorter overall survival (hazards ratio [HR] = 2.390, confidence interval [CI] = 0.9259–6.1680, *p* = 0.0247; Fig. 1c) and recurrence-free survival (HR = 1.746, CI = 1.123–2.717, *p* = 0.0022; Supplementary Fig. S1c). Analysis of βPix expression using 60 cell lines derived from different cancer tissues in the GEO databases revealed that βPix expression was highest in cells derived from CRC tissues (Supplementary Fig. S1d). Furthermore, we found that high levels of βPix expression in CRC cell lines corresponded to increased invasive and migrative abilities (Fig. 1d; Supplementary Fig. S1e).

To determine whether the level of βPix affected CRC cell progression, including 3D sphere formation, migration and invasion, we knocked down and overexpressed the *βPix* gene in SW480 and LoVo cells, which endogenously express βPix protein at relatively high and low levels, respectively (Supplementary Fig. S1f and h). Compared with control cells, SW480 cells with *βPix* silencing showed a significantly decreased persistence and velocity of single-cell motility, as well as inhibited 3D sphere formation (Fig. 1e–g; Supplementary Video S1), whereas *βPix* overexpression in LoVo cells promoted migration, as observed in the wound healing assay (Supplementary Fig. S1i). In addition, depletion of *βPix* in SW480 cells significantly inhibited their invasive activity, which was associated with downregulated MMP activity regardless of MT1-MMP expression (Fig. 1h; Supplementary Fig. S1f and g). Similarly, CRC cell invasion was increased following *βPix* overexpression in LoVo cells (Supplementary Fig. S1j). These results indicate that βPix is required for CRC tumorigenicity and invasion.

### βPix promotes invasive migration via lamellipodial localization of MT1-MMP

**Fig. 2:**
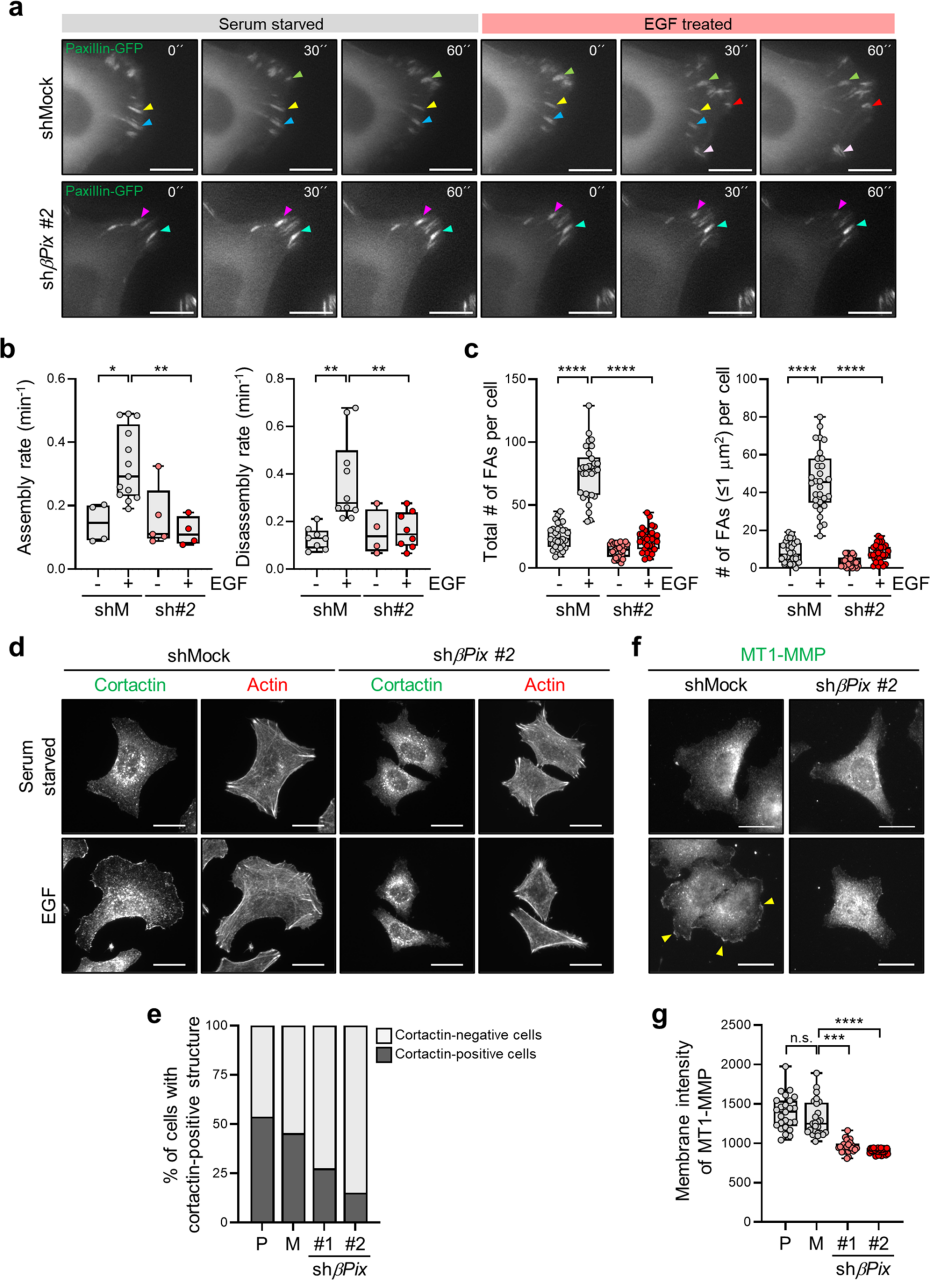
βPix facilitates lamellipodia formation and membrane localization of MT1-MMP, leading to increased invasive migration. **a** Representative images from the time-lapse video microscopy of paxillin-GFP-expressing shMock and sh*βPix* #2SW480 cells under EGF stimulation, monitored for 1 h at 2-min intervals. Colored arrowheads indicate dynamic and stable focal adhesions (FAs), respectively. Scale bars, 10 μm. **b** FA assembly (left) and disassembly (right) rates from shMock and sh*βPix* #2 SW480 cells under EGF stimulation. Statistical analysis was performed using one-way ANOVA with Tukey’s multiple comparisons from three independent experiments. One-way ANOVA, F_3, 22_ = 8.038 and F_3, 26_ = 7.900. **c** The total number of FAs (left) and nascent FAs under 1 µm^2^ (right) in shMock and sh*βPix* #2 SW480 cells within a 10-µm region from the leading edge upon EGF treatment (n = 30, each group). One-way ANOVA, F_3, 116_ = 145.5 and F_3, 116_ = 147.6. **d** Representative images of cortactin and F-actin stained with Phalloidin-594 in shMock and sh*βPix* #2 SW480 cells under EGF treatment. Scale bars, 20 μm. **e** Bar graph representing the percentage of EGF-induced shMock and sh*βPix* #2 SW480 cells with lamellipodia structure. **f** Immunofluorescence imaging of MT1-MMP in shMock and sh*βPix* #2 SW480 cells under EGF stimulation. Arrowheads indicate membrane-localized MT1-MMP. Scale bar, 20 μm. **g** Fluorescence intensity of MT1-MMP at the membrane edge of shMock and sh*βPix* #2 SW480 cells (n = 25, each group). One-way ANOVA, F_3, 96_ = 56.06. *p < 0.05, **p < 0.01, ***p < 0.001, ****p < 0.0001, n.s. not significant; EGF, epidermal growth factor; MT1-MMP, membrane-type 1 matrix metalloproteinase

βPix is well-known for regulating membrane dynamics through Rac1 small GTPase-mediated actin rearrangement during cell migration [32, 33]. Accordingly, to examine whether CRC cell invasion is dependent on the βPix-mediated process, we first measured FA dynamics at the leading edge of shMock and *βPix*-silenced SW480 cells using time-lapse video microscopy. Given that EGF stimulation promotes FA dynamics in migrating cells, we monitored FA dynamics using paxillin-GFP under EGF stimulation. Following EGF stimulation, the newly formed FAs at the leading edge were markedly inhibited in *βPix-*silenced SW480 cells, resulting in significantly reduced FA assembly and disassembly rates (Fig. 2a and b; Supplementary Video S2). In particular, *βPix*-silenced cells exhibited a decreased number of small nascent FAs (areas under 1 mm^2^), as well as a reduction in the total number of FAs at the leading edge (Fig. 2c; Supplementary Fig. S2a). The EGF-induced lamellipodial formation was also suppressed in *βPix*-silenced cells (Supplementary Fig. S2b). Immunostaining for cortactin, a lamellipodia marker, revealed that *βPix* depletion significantly decreased the extension of membrane protrusions (Fig. 2d and e). By contrast, overexpression of Flag-*βPix* in LoVo cells enhanced cell migration, and longer membrane protrusions were observed in the direction of migration (Supplementary Fig. S2c). Surprisingly, upon EGF stimulation, MT1-MMP, which is responsible for extracellular matrix (ECM) degradation during cell invasion, relocated to the membrane protrusion area in shMock cells. In *βPix-*silenced cells, MT1-MMP localization to the membrane periphery was impaired (Fig. 2f and g). Collectively, our findings indicate that βPix potentiates the invasive migration of CRC cells by promoting actin cytoskeleton remodeling and lamellipodial localization of MT1-MMP.

### βPix binds to Dyn2 at the leading edge of cells via its SH3 domain

**Fig. 3:**
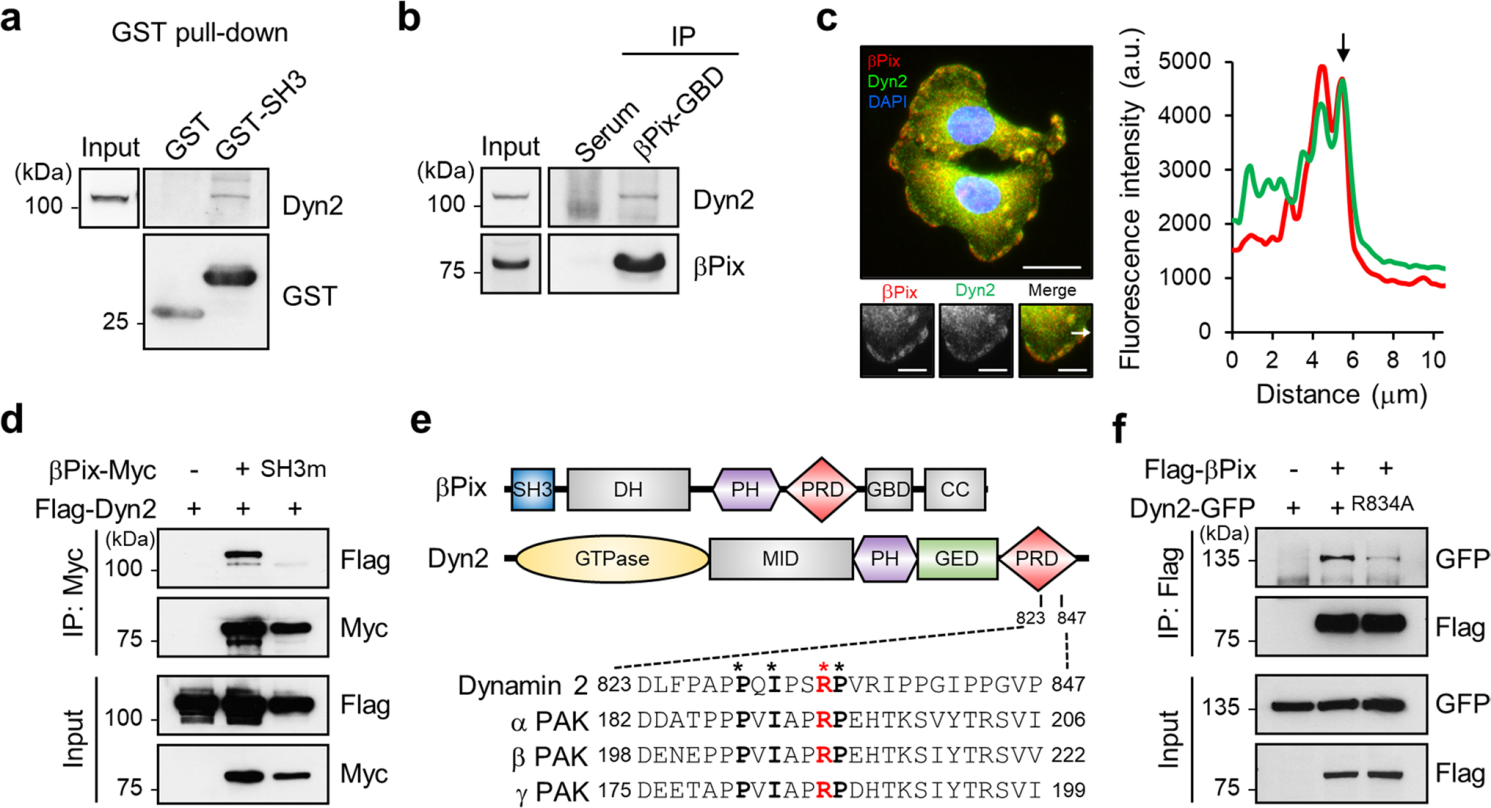
SH3 domain of βPix interacts with PRD of Dyn2 at the membrane edge.** a** Pull-down assay using HEK293T cell lysates with GST or GST-SH3 proteins. Dyn2 was detected using Western blotting. **b** Immunoprecipitation of endogenous Dyn2 with anti-βPix-GBD antibodies specifically targeting GBD of βPix. **c** Double staining of βPix and Dyn2 in SW480 cells using fluorescein-conjugated antibodies under FBS conditions. The fluorescence line intensity was measured along the direction of the white arrow. The black arrow in the graph indicates the membrane ruffle. Scale bars, 20 μm (upper image) and 5 μm (lower images). **d** Immunoprecipitation assay of HEK293T cells transfected with βPix-Myc or βPix-SH3 mutant-Myc and Flag-Dyn2 using anti-Myc antibodies. **e** Schematic representation of the βPix and Dyn2 domains with sequence alignment of the conserved proline-rich βPix-binding sequence in PAK isoforms and Dyn2. Residues completely conserved are in bold (important motif for Pix binding). **f** Immunoprecipitation with anti-Flag antibodies to verify the interaction of Flag-βPix with Dyn2-GFP or Dyn2 R834A-GFP after transfection with Flag-βPix and Dyn2-GFP or Dyn2 R834A-GFP in HEK293T cells. SH3, Src homology 3; DH, Dbl homology; PH, pleckstrin homology; PRD, proline-rich domain; GBD, GTPase-binding domain; CC, coiled-coil region; MID, middle domain; GED, GTPase effector domain

βPix is a well-known GEF that activates Rac1, and its GEF activity increases upon interacting with other binding proteins via its SH3 domain [6]. Accordingly, we attempted to identify a potential binding partner of βPix involved in CRC cell migration and invasion by proteomics via a GST pull-down assay using GST-βPix-SH3 purified protein. Proteins interacting with the GST-βPix-SH3 protein were visualized by silver staining and subjected to MALDI-TOF analysis (data not shown). Dyn2 was identified as the major protein interacting with the SH3 domain of βPix based on proteomic analysis. GST pull-down and immunoprecipitation experiments with anti-βPix antibody confirmed the interaction between βPix and Dyn2 (Fig. 3a and b). Furthermore, fluorescence images of stained endogenous βPix and Dyn2 revealed that they colocalize at the plasma membrane periphery, supporting the results of the pull-down analysis (Fig. 3c). Functional mutation of the SH3 domain in βPix (substitution of *Trp* at position 43 of *Lys*; [8]) impaired binding to Dyn2 (Fig. 3d), indicating that the SH3 domain participates in the interaction between βPix and Dyn2.

As the SH3 domain is known to be involved in protein binding through the PRD of other proteins [7], we examined whether the PRD of Dyn2, which is relatively similar to that of PAK isoforms (Fig. 3e), interacts with the SH3 domain of βPix. Mutation of *Arg* 834 to *Ala* in the PRD of Dyn2, which reportedly induces defective binding with the SH3 domain [7], significantly decreased the βPix interaction with Dyn2 (Fig. 3f). Together, these results suggest that the PRD of Dyn2 binds to the SH3 domain of βPix.

### The βPix-Dyn2 complex enhances Rac1-mediated lamellipodia formation

**Fig. 4:**
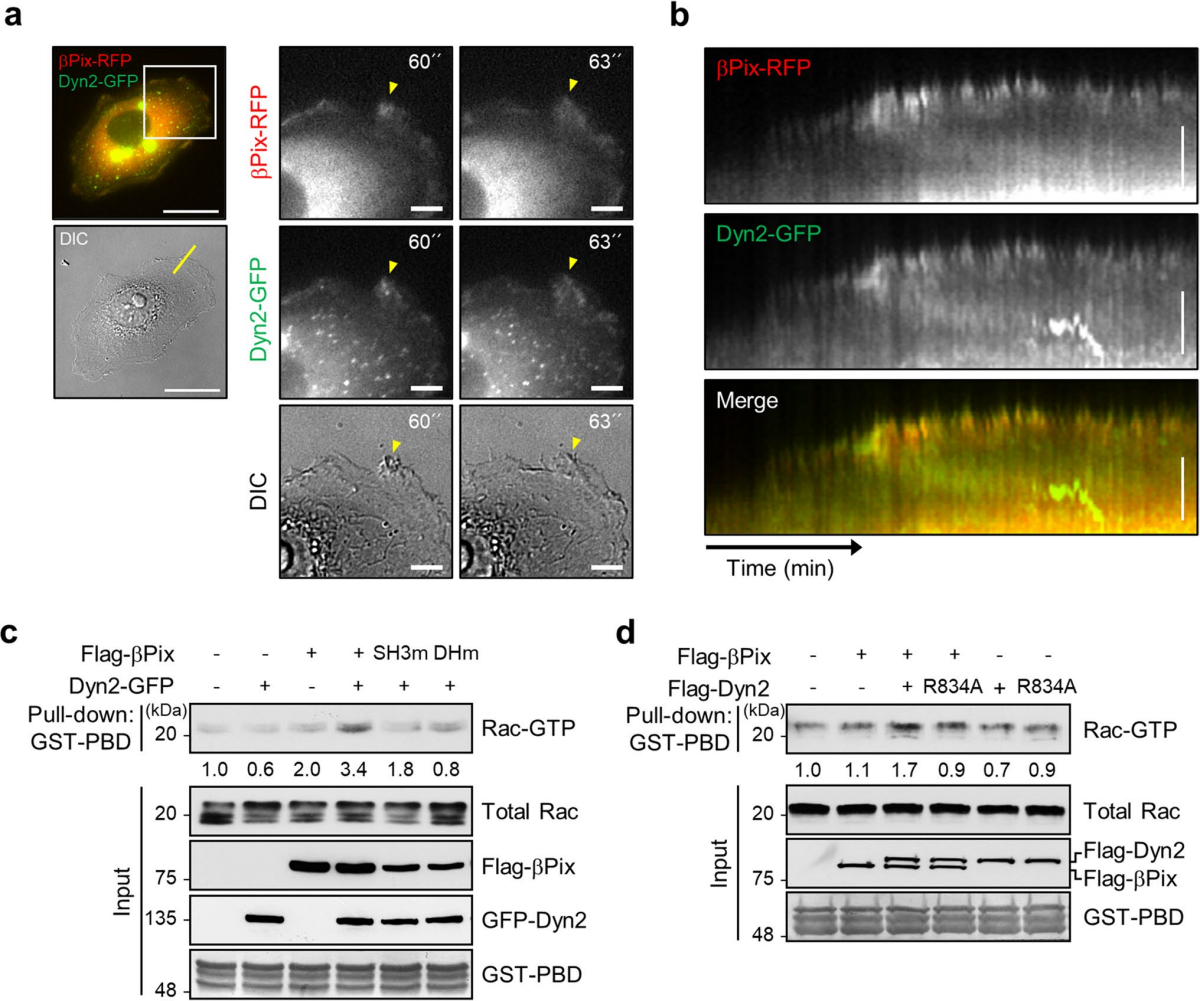
The βPix-Dyn2 complex induces active Rac1-mediated membrane ruffling.** a** Time-lapse images of SW480 cells co-transfected with βPix-RFP and Dyn2-GFP under epidermal growth factor (EGF) stimulation. Cropped time-lapse images are presented from the boxed region. Yellow arrowheads represent the position of colocalization and membrane ruffles. Scale bar, 20 μm (left images) and 5 μm (right images). **b** Kymograph analysis of the membrane edge in SW480 cell expressing βPix-RFP and Dyn2-GFP using time-lapse imaging for 3 h at 1-min intervals. Kymographs were created from the lined region shown in **a**. **c** GST-PBD pull-down assay of Rac1 activity in HEK293T cells transfected with Flag-βPix, SH3 or DH with/without Dyn2-GFP. Rac1 activity was estimated by normalizing the band intensity of Rac1-GTP against that of total Rac1. The ratio below indicates the relative Rac1 activity compared with non-transfected cells from four independent experiments. **d** GST-PBD pull-down assay in HEK293T cells transfected with Dyn2-GFP or Dyn2-R834A-GFP with/without Flag-βPix. The ratio below indicates the relative Rac1 activity normalized by non-transfected cells from three independent experiments

Reportedly, Dyn2 assists the GEF activity of Vav1 to increase lamellipodia formation and cell migration by regulating Vav1 stability [34]. Accordingly, we investigated whether Dyn2 influences the GEF activity of βPix, leading to Rac1 activation and lamellipodia formation. Time-lapse microscopy (Fig. 4a) showed marked colocalization between βPix-RFP and Dyn2-GFP on lamellipodia and induced membrane ruffling at the leading edge. Furthermore, kymograph analysis from time-lapse microscopy indicated that colocalization of βPix-RFP and Dyn2-GFP was enriched on the membrane edge, given the increased membrane ruffling (Fig. 4b; Supplementary Video S3). These results indicate that interaction of βPix and Dyn2 at the membrane periphery is required to promote membrane ruffling.

As membrane ruffing is induced by Rac1 and βPix is known to be a GEF for Rac1, we hypothesized that Dyn2 facilitates the GEF activity of βPix to induce Rac1-mediated membrane ruffling. To test this hypothesis, we performed Rac1 activity assessment using a GST-PBD pull-down assay with various βPix and Dyn2 mutants. We found that βPix alone resulted in a two-fold increase in Rac1 activation, whereas Dyn2 alone minimally impacted Rac1 activation (Fig. 4c; lanes 1–3). Interestingly, overexpression of both βPix and Dyn2 increased Rac1 activation by approximately three-fold. However, a SH3 mutant of βPix, known to impair the βPix-Dyn2 complex, decreased Rac1 activation by 50 %, similar to that of the functional βPix mutant (DH domain mutation) (Fig. 4c; lanes 4–6). In addition, a R834A mutant of Dyn2, which is deficient in βPix binding, decreased Rac1 activity (Fig. 4d). These results indicate that Dyn2 plays an important role in βPix-mediated Rac1 activation.

### Dyn2 is required for βPix-mediated lamellipodial formation and MT1-MMP localization at the membrane periphery

**Fig. 5:**
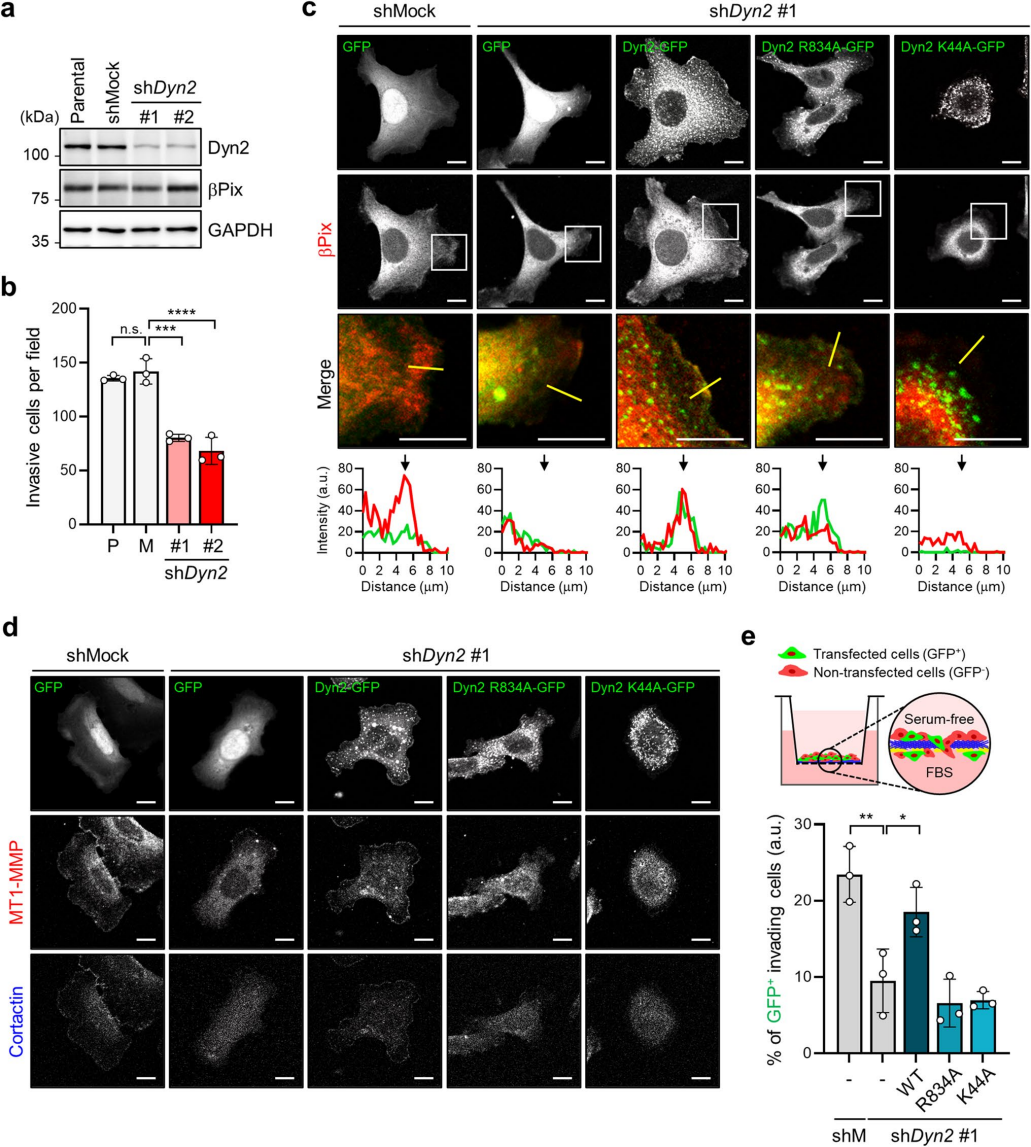
Dyn2 mediates βPix-induced lamellipodia formation and MT1-MMP localization at the membrane edge, thus promoting cell invasion. **a** Generation of sh*Dyn2* SW480 cell lines with different shRNA oligos and verification of downregulated Dyn2 protein levels using Western blotting. GAPDH was used as a loading control. **b** The number of invasive sh*Dyn2* SW480 cells was evaluated by randomly imaging cells stained with crystal violet at the bottom of the Transwell. Data are presented as the mean ± standard deviation (S.D.) from three independent experiments. Statistical analysis was performed using one-way ANOVA with Tukey’s multiple comparisons. One-way ANOVA, F_3, 8_ = 54.63. **c** Immunofluorescence images of βPix in shMock and sh*Dyn2* #1 SW480 cells transfected with the indicated vectors under EGF stimulation for 10 min. Merge shows the enlarged images from the boxed regions. The fluorescence line intensity was analyzed along the white line, and black arrows indicate the membrane edge. Scale bar, 20 μm. **d** Immunofluorescence images of MT1-MMP and cortactin in shMock and sh*Dyn2* #1 SW480 cells transfected with the indicated vectors under EGF stimulation for 10 min. Scale bar, 20 μm. **e** Transwell assay of GFP-positive invasive cells with shMock and sh*Dyn2* #1 expression vectors under serum condition. The percentage of GFP-positive invasive cells was calculated by dividing GFP^+^ cells in the lower chamber by total GFP^+^ cells. Data are presented as the mean ± S.D. from three independent experiments. One-way ANOVA, F_4, 10_ = 16.43. *p < 0.05, **p < 0.01, ***p < 0.001, ****p < 0.0001, n.s. not significant; EGF, epidermal growth factor; MT1-MMP, membrane-type 1 matrix metalloproteinase

To further clarify the involvement of Dyn2 in βPix-induced lamellipodial formation and cell invasion, we generated *Dyn2*-silenced SW480 cells with two individual shRNA oligos (Fig. 5a). Initially, we examined whether Dyn2 regulates βPix protein stability for Rac1 activation, as previously reported [34]. We found that in *Dyn2*-silenced SW480 cells the protein level of βPix was unaltered, and *vice versa* (Fig. 5a; Supplementary Fig. S3a), indicating that Dyn2 does not affect the stability of βPix.

Similar to the characteristics of the *βPix*-knockdown cells shown in Fig. 1e and h, invasion and 3D sphere formation were significantly reduced in *Dyn2*-silenced cells (Fig. 5b; Supplementary Fig. S3b). Additionally, upon EGF stimulation, lamellipodial formation and FA dynamics were suppressed in *Dyn2*-silenced cells, although the βPix level was normal (Supplementary Fig. S3c–e). This suggests that the interaction between Dyn2 and βPix may induce cell invasion and sphere formation in CRC cells. Interestingly, the reduced membrane localization of βPix in *Dyn2*-silenced cells was restored by overexpression of wild-type *Dyn2*. Although the Dyn2 R834A mutant was observed at the membrane periphery, it did not induce βPix localization at this site (Fig. 5c). Moreover, Dyn2 K44A, which is deficient in GTPase activity, did not recruit βPix to the membrane periphery (Fig. 5c), indicating that the enzyme activity of Dyn2 may impact the function of βPix in cell invasion.

Next, we examined whether MT1-MMP localization on lamellipodia was dependent on βPix-Dyn2 complexes. We found that the localization of lamellipodial MT1-MMP was inhibited in *Dyn2*-silenced cells, and that overexpression of the Dyn2 PRD mutant (R834A) or GTPase-deficient mutant (K44A), except for the wild-type, failed to restore the peripheral localization of MT1-MMP (Fig. 5d). Consistent with this finding, invasion of *Dyn2*-silenced cells was not restored by R834A or K44A Dyn2 mutants, except for wild-type Dyn2 (Fig. 5e). Furthermore, we performed kymograph analysis of LifeAct-RFP to monitor the membrane extension in *βPix-*overexpressing *Dyn2*-silenced SW480 cells following EGF stimulation. We detected a marked decrease in membrane extension and cell invasion, despite *βPix* overexpression (Supplementary Fig. S3f and g; Supplementary Video S4). These results indicate that Dyn2 is required for the membrane localization of βPix, mediated by interaction with the Dyn2 PRD and the enzymatic activity of Dyn2. Thus, the βPix-Dyn2 complex enhances lamellipodia formation and MT1-MMP localization at the membrane periphery, resulting in increased cell invasion.

### 3.6 The βPix-Dyn2 complex is enhanced by Src-induced βPix Y442 phosphorylation

**Fig. 6:**
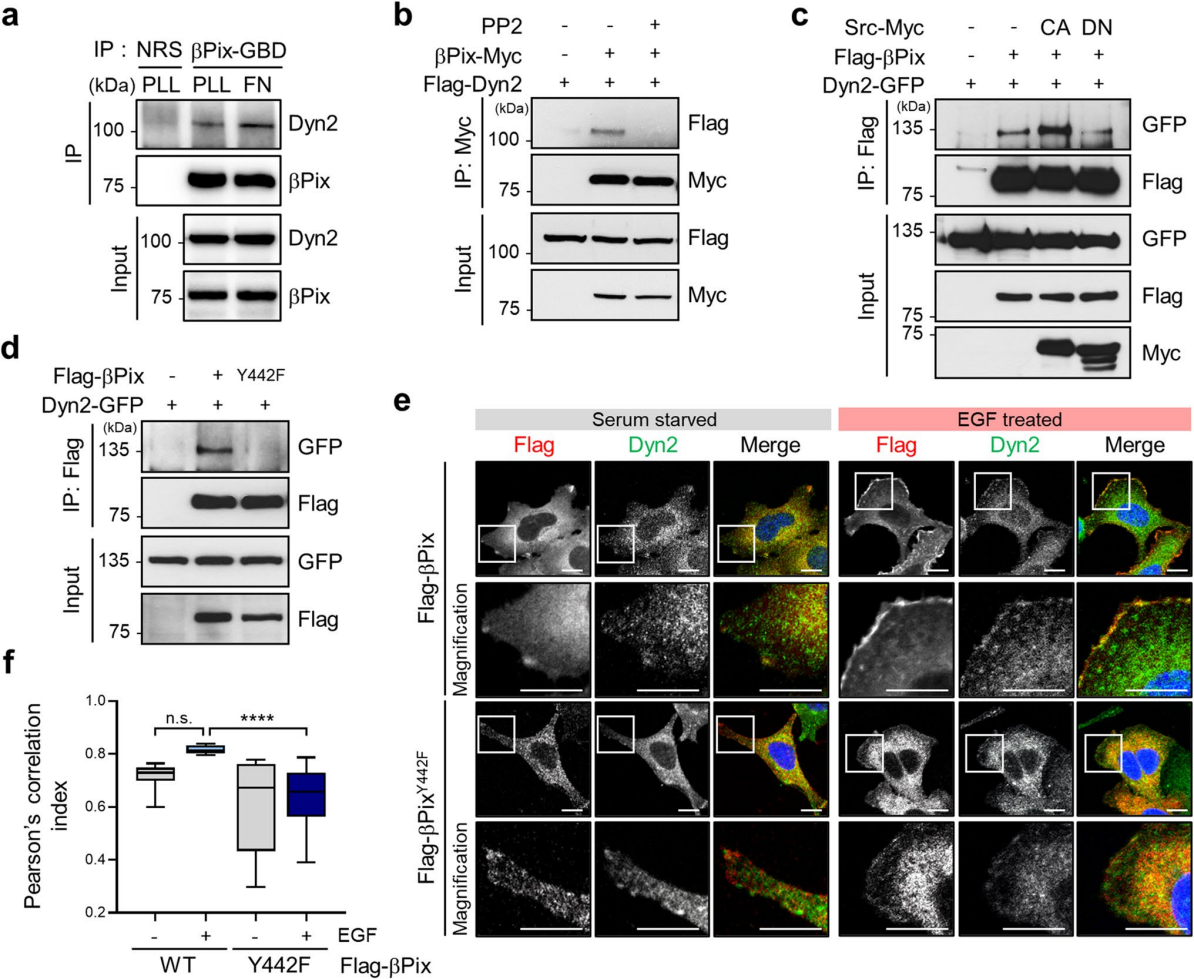
Src-mediated βPix phosphorylation enhances βPix-Dyn2 complex formation.** a** HEK293T cells were seeded on poly-L-lysine or fibronectin-coated dishes for 15 min after 1 h in suspension. Cell lysates were subjected to immunoprecipitation with anti-βPix-GBD antibodies. **b** HEK293T cells transiently expressing Flag-βPix and Dyn2-GFP were treated with 5 µM PP2 for 30 min and subjected to immunoprecipitation. **c** and **d** Immunoprecipitation with anti-Flag antibodies in HEK293T cells transfected with the indicated vectors. **e** Immunofluorescence images of endogenous Dyn2 with Flag-tagged βPix and βPix Y442F in SW480 cells under EGF stimulation for 10 min. Magnifications represent enlarged images from the boxed regions. Scale bar, 20 μm. **f** Pearson’s correlation index was measured to quantify the colocalization of Dyn2 and Flag-βPix WT or Y442F (n = 15, each group). Statistical analysis was performed using one-way ANOVA with Tukey’s multiple comparisons. One-way ANOVA, F_3, 56_ = 11.22. ****p < 0.0001, n. s. not significant; NRS, normal rabbit serum; CA, constitutively active Src; DN, dominant-negative Src; EGF, epidermal growth factor

Tumor cell invasion predominantly requires adhesion to the ECM via cell surface receptors, such as integrins [35, 36]. Therefore, we next determined whether βPix and Dyn2 binding was dependent on cell-matrix adhesion. To this end, immunoprecipitation experiments for βPix and Dyn2 interactions were performed using cells incubated under poly L-lysine-coated or fibronectin-coated culture conditions, respectively. Interestingly, the interaction of βPix and Dyn2 was enhanced under fibronectin conditions, where FA and lamellipodia formation was markedly enhanced (Fig. 6a; Supplementary Fig. S4a). This interaction was significantly inhibited following treatment with PP2, a selective inhibitor of Src family kinases (Fig. 6b). Moreover, we found that constitutive activation of Src kinase facilitated the interaction between βPix and Dyn2. Conversely, overexpression of a dominant-negative mutant of Src kinase suppressed the interaction between βPix and Dyn2 (Fig. 6c). These findings indicate that under fibronectin culture conditions, activation of Src kinase facilitates formation of the βPix-Dyn2 complex.

Reportedly, phosphorylation of tyrosine residues at position 442 by Src kinase contributes to the regulation of protein interactions [37]. Thus, we next examined whether Src-induced phosphorylation of tyrosine at position 442 (Y442) in βPix is critical for interacting with Dyn2. We first demonstrated that Y442 in βPix was phosphorylated by constitutively activated Src kinase, which was incapable of phosphorylating a βPix mutant (Y442F; Supplementary Fig. S4b), as shown in a previous study [38]. Notably, the Y442F mutant of βPix showed decreased interaction with Dyn2 (Fig. 6d). Additional immunofluorescence imaging revealed that βPix Y442F sparsely colocalized with endogenous Dyn2 at the membrane periphery compared with wild-type βPix (Fig. 6e). Consistent with this finding, colocalization in lamellipodia regions, quantified using Pearson’s coefficients, was significantly reduced in the βPix Y442F-expressing mutant (Fig. 6f). However, Y442E, a phosphorylation-mimicking mutant, restored membrane colocalization of βPix and Dyn2 (Supplementary Fig. S4c), indicating that phosphorylation of Y442 in βPix facilitated its interaction with Dyn2.

Next, we investigated whether the invasiveness of CRC cells was affected by the status of βPix Y442 phosphorylation using a Transwell assay. We found that βPix shRNA targeting its 3′UTR downregulated CRC cell invasion, which was subsequently restored by overexpression of wild-type βPix or βPix Y442E mutant. However, exogenous expression of the βPix Y442F mutant failed to reverse the suppressed invasion (Supplementary Fig. S4d). Collectively, these results indicate that Src-induced phosphorylation of the tyrosine residue at position 442 in βPix is essential for βPix-Dyn2 interaction at the leading edge of the membrane, thus facilitating cell invasion.

### Disrupting the βPix-Dyn2 interaction impairs membrane dynamics and cancer cell invasion

**Fig. 7:**
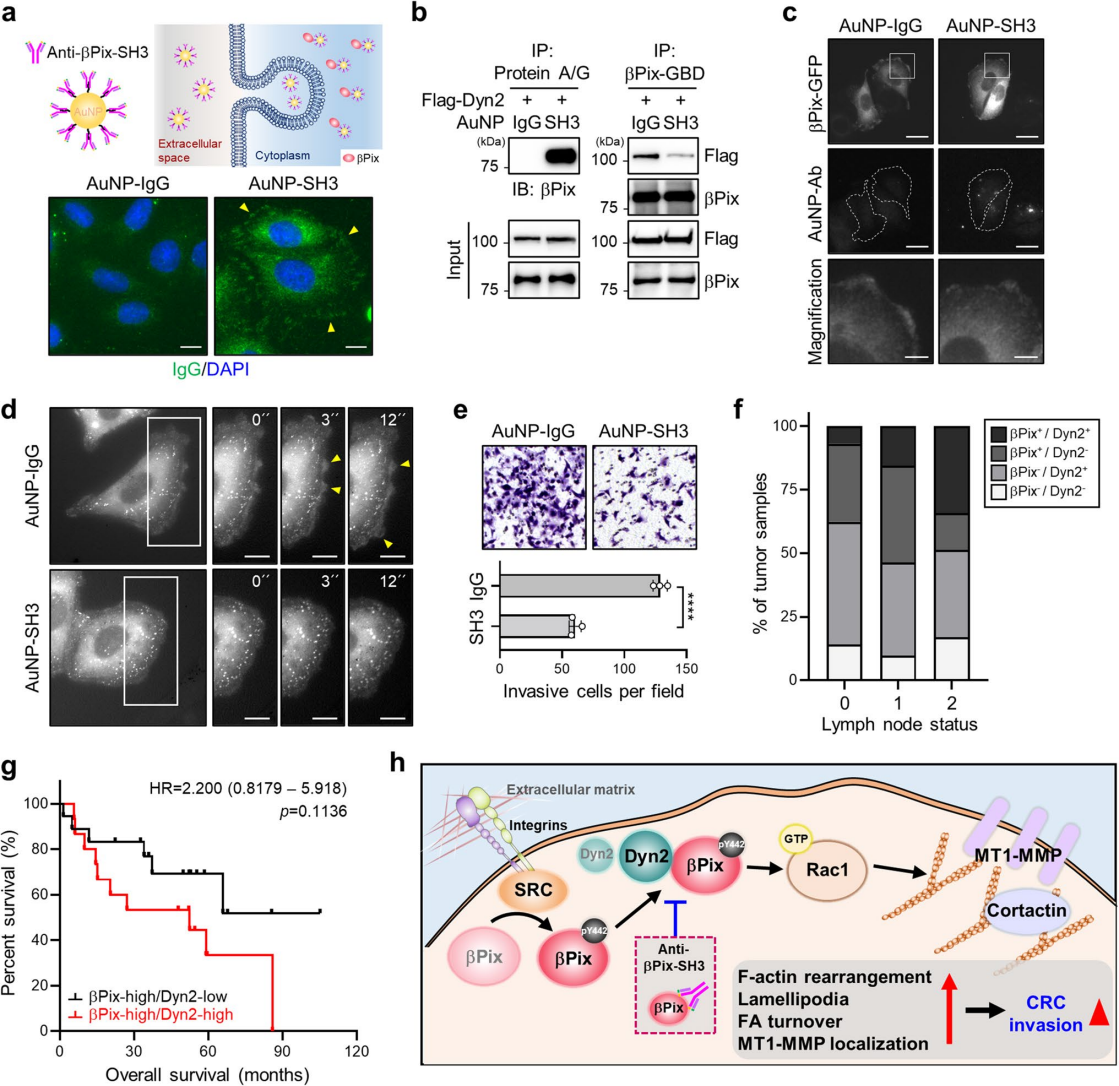
AuNP-SH3 inhibits cell invasion by disrupting the interaction between βPix and Dyn2. **a** Schematic representation of the generated AuNP-SH3 conjugates. The fluorescent images below show the internalized AuNP-antibody conjugates (green) and nuclei (blue) in SW480 cells. Arrowheads indicate endogenous βPix signals. Scale bars, 5 μm. **b** Immunoprecipitation with protein A/G agarose beads (left panel) and anti-βPix-GBD antibodies (right panel) in SW480 cells expressing Flag-Dyn2 treated with AuNP-IgG or AuNP-SH3. **c** Representative images of βPix-GFP-expressing SW480 cells with AuNP-IgG or AuNP-SH3 following EGF stimulation for 20 min. Dotted lines indicate cells treated with AuNP-antibody conjugates, and cropped images show the membrane localization of βPix-GFP. Scale bars, 20 and 5 μm (magnified images). **d** Time-lapse images of Dyn2-GFP transfected SW480 cells treated with AuNP-IgG or AuNP-SH3 following EGF stimulation, monitored for 30 min at 1-min intervals. Cropped images from boxed regions show magnifications of the membrane edges, indicating membrane-localized Dyn2-GFP and ruffles with yellow arrowheads. Scale bar, 10 μm. **e** Invasive activities of AuNP-IgG- or AuNP-SH3-treated SW480 cells using Transwell assays. The average number of invasive cells per field was calculated. Data are presented as the mean ± S.D. from three independent experiments. Statistical analysis was performed by Student’s unpaired t-test. **f** Stacked bar plots representing the distribution of βPix-Dyn2 expression in patients with CRC across three lymph node stages from TCGA datasets. **g** Kaplan-Meier analysis of the overall survival of patients with CRC (33 patients) with a high level of Dyn2 among patients with βPix overexpression using the GSE29621 dataset. **h** Proposed model for βPix-Dyn2 complex-mediated CRC cell invasion via modulation of the membrane dynamics and MT1-MMP localization. Dyn2 was identified as a novel binding partner of βPix, one of the most well-known tumor-promoting factors. The phosphorylation of tyrosine 442 in βPix by Src kinase promotes interaction with Dyn2. The βPix-Dyn2 complex then activates Rac1 and induces membrane dynamics, resulting in increased CRC cell invasion. The disruption of the βPix-Dyn2 complex by βPix-SH3 antibody-conjugated AuNPs decreases membrane ruffles and CRC cell invasion. ****p < 0.0001; EGF, epidermal growth factor; CRC, colorectal cancer; MT1-MMP, membrane-type 1 matrix metalloproteinase; TCGA, The Cancer Genome Atlas

Our results revealed that the βPix-Dyn2 complex in CRC cells is required for lamellipodia formation and invasion. To verify a crucial role of the βPix-Dyn2 interaction in regulating membrane dynamics, we utilized a βPix-SH3 antibody-conjugated AuNP delivery system for disrupting the interaction between βPix-Dyn2 by targeting the SH3 domain of βPix (Fig. 7a; Supplementary Fig. S5a). Fluorescence imaging of SW480 cells incubated with AuNP-SH3 showed strong green fluorescence signals as dot-like structures near the peripheral area, unlike the cells treated with AuNP-IgG with no signals. This indicates that AuNP-SH3 may specifically target endogenous βPix (Fig. 7a). Additional immunoprecipitation assays using only protein A/G agarose beads revealed that the internalized AuNP-SH3 targeted βPix in Flag-Dyn2-expressing SW480 cells (Fig. 7b; left panel), whereas immunoprecipitation with anti-βPix-SH3 antibody showed that the interaction of the βPix-Dyn2 complex was significantly decreased by AuNP-SH3 (Fig. 7b; right panel). After EGF stimulation, the membrane localization of βPix was reduced by AuNP-SH3 delivery (Fig. 7c). To verify whether AuNP-SH3 interrupts the cellular function of the βPix-Dyn2 complex, we monitored membrane ruffling in Dyn2-GFP-expressing SW480 cells treated with AuNP-SH3 (Fig. 7d). Time-lapse video imaging showed reduced membrane ruffles in AuNP-SH3-treated SW480 cells when compared with active membrane ruffles enriched with Dyn2 in AuNP-IgG-treated cells (Fig. 7d; Supplementary Video S5). These results indicate that the βPix-Dyn2 interaction is essential for downstream effects. We next investigated whether disruption of the βPix-Dyn2 complex by AuNP-SH3 inhibited cell invasion. As expected, SW480 cells treated with AuNP-SH3 showed reduced invasiveness compared with cells treated with AuNP-IgG (Fig. 7e). These findings suggest that interfering with the βPix-Dyn2 interaction exerts antitumor effects by inhibiting CRC cell invasion.

We further aimed to establish the correlation between βPix and Dyn2 expression to support the clinical relevance of the βPix-Dyn2 complex in primary CRC samples. Unexpectedly, Dyn2 expression did not differ between normal and CRC tissues in the Oncomine database, where βPix expression was upregulated in CRC tissues, as shown in Fig. 1a (Supplementary Fig. S5b). In addition, Dyn2 transcript levels showed no correlation with the βPix level, as indicated by the data from TCGA database (Supplementary Fig. S5c). However, patients with CRC and high βPix and Dyn2 expression exhibited significantly increased rates of lymph node metastasis (Fig. 7f). According to the survival analysis of patients with CRC and elevated βPix expression, increased Dyn2 expression was closely associated with low survival (Fig. 7g). Collectively, we conclude that a high level of the βPix-Dyn2 complex can predict a poor prognosis in patients with CRC, suggesting its potential application as a combinatorial marker for targeted therapy.

## Discussion

In the present study, we show that a spatiotemporal regulation of the βPix-Dyn2 interaction, by cell-matrix adhesion-mediated Src kinase activation, is crucial for CRC cell invasion. The βPix-Dyn2 complex allowed cells to increase lamellipodia formation by promoting the GEF activity of βPix to Rac1. Consequently, this process induced MT1-MMP recruitment to the leading edge of the cell membrane periphery, followed by increased invasive activity (Fig. 7h).

The function of βPix as a GEF protein for small Rho GTPases is determined by the interaction of binding proteins owing to its relatively weak GEF activity [39]. Accordingly, various proteins that bind to the SH3 domain of βPix have been identified as cellular function regulators of βPix. For example, E3 ligase Cbl competitively binds to the SH3 domain of βPix with Pak1 to simultaneously regulate Cdc42 and EGFR signaling [40]. In addition, βPix recruitment to the leading edge by interacting with scaffold proteins, such as Scrib and STIL, has been found to control the spatial regulation of membrane dynamics during cell migration and cell polarization [41–43]. Here, we identified Dyn2 as a novel binding protein regulating the GEF activity of βPix. The molecular mechanism through which binding to Dyn2 increases the GEF activity of βPix remains unclear, but when *βPix* is overexpressed in cells with inhibited *Dyn2* expression, membrane localization of βPix does not occur and βPix-dependent lamellipodia formation is suppressed. In addition, lamellipodia formation did not occur despite *Dyn2* overexpression in *βPix-*silenced cells (data not shown). Therefore, the spatial interaction of βPix and Dyn2 near the plasma membrane likely provides the driving force for increasing the GEF activity of βPix, resulting in active βPix-induced Rac1 and increased membrane dynamics.

On the basis of our observations, disrupting the βPix-Dyn2 interaction using the Dyn2 R834A mutant inhibited Rac1 activation, lamellipodia formation and cell invasion, although the enzymatic activity of Dyn2 was unaltered. However, Dyn2 K44A, a mutant lacking enzymatic activity, also reduced βPix recruitment at the membrane edge, decreasing lamellipodia formation and invasion. Interestingly, immunoprecipitation assays revealed that βPix continuously interacted with the Dyn2 K44A mutant (data not shown), suggesting that the GTPase activity of Dyn2 is also required for the GEF activity of βPix. Dyn2 has been proposed as a well-defined mechanoenzyme that drives membrane fission from the plasma membrane and clathrin-mediated endocytosis at endocytic sites of the plasma membrane [25, 44]. These Dyn2 functions are largely mediated by GTPase activity, as the GTPase-deficient Dyn2 K44A mutant results in impaired endocytosis and membrane trafficking with abnormal cellular distribution [45, 46]. In the case of Dyn2-mediated Rac1 modulation, Dyn2 is reportedly involved in Rac1 internalization into macropinosomes, as well as Rac1 recycling into integrin at the membrane edge, where lamellipodia formation is increased via the GTPase activity of Dyn2 during cell migration [47, 48]. However, dominant-negative Dyn2 (Dyn2 K44A) increases total Rac1 activity by inhibiting Rac1 internalization, thus accumulating active Rac1 in abnormal membrane positions and decreasing Rac1 trafficking toward newly generated lamellipodia [48]. Our results also revealed that cellular localization of Dyn2 K44A could be observed throughout the cytoplasm with reduced membrane localization in SW480 cells while maintaining the interaction with βPix. Therefore, we postulate that if βPix binds to Dyn2, βPix can activate Rac regardless of its cellular localization. Interestingly, the GTPase activity of Dyn2 is critical for membrane targeting of the βPix-Dyn2 complex, and βPix-mediated Rac1 activation at the membrane edge is required for lamellipodia formation and CRC cell invasion. Our results indicate that spatially regulated membrane localization of the βPix-Dyn2 complex via Dyn2 GTPase activity is required for Rac1-dependent membrane dynamics during CRC cell invasion.

Furthermore, our results revealed that *βPix* knockdown decreased MT1-MMP localization in the membrane periphery and inhibited lamellipodia formation in CRC cells. Reportedly, actin rearrangement for invasive migration increases the concentration of MT1-MMP in lamellipodia [49]. In HT1080 fibrosarcoma cells in fibril gel, the expression of MT1-MMP was found to be induced by active Rac1, which in turn was induced by GEF proteins [50]. Furthermore, βPix has been found to promote Rac3 activity in serous ovarian cancer by forming a complex with β-arrestin1/integrin-linked kinase, thus supporting MT1-MMP-dependent ECM degradation at invadopodia formation [51, 52]. Thus, it can be speculated that βPix-mediated Rac1 activation may be required for CRC cell invasion to promote actin rearrangement, lamellipodia formation, and MT1-MMP trafficking at the peripheral area of the leading edge. Additionally, we found that the downregulated Rac1 activity caused by a disrupted βPix-Dyn2 interaction via Dyn2 PRD mutant reduced lamellipodial localization of MT1-MMP and CRC cell invasion. This finding indicates that the βPix-Dyn2 complex is essential for MT1-MMP recruitment toward the leading edge of invasive CRC cells in a Rac1-dependent manner.

During metastatic progression, tumor cells require fine-tuned phases of metastatic cascades that can sense the surrounding tumor microenvironment, degrade the ECM, and migrate through the processed matrix [53]. Cell-ECM adhesion-dependent signals, including integrins and growth factors, temporally coordinate the activation of downstream signaling pathway factors, such as Src family kinases, FA kinase (FAK), ERK and PI3K/AKT, for tumor cell invasion [54, 55]. Thus, we hypothesize that the formation of the βPix-Dyn2 complex is temporally regulated under cell adhesion conditions. It has been reported that βPix is phosphorylated by Src, FAK and PAK2, and then activated and recruited at lamellipodia, thus promoting downstream signaling in several model systems [38, 56]. In particular, phosphorylation of the tyrosine 442 residue of βPix has been shown to enhance its GEF activity of Cdc42 and promote the formation of βPix-Cdc42-Cbl complex to suppress EGFR degradation [38, 40]. Furthermore, constitutive phosphorylation of βPix Y442 in v-Src-expressing fibroblasts interferes with EGFR homeostasis and causes cell transformation, tumorigenesis, migration and invasion in *in vivo* systems [37]. In line with the importance of βPix Y442 phosphorylation in tumor progression, we also investigated whether Src kinase-induced βPix Y442 phosphorylation modulated the invasive activity of CRC cells by temporally regulating the formation of the βPix-Dyn2 complex. How was the βPix-Dyn2 interaction temporally controlled for βPix-mediated CRC invasion? One possible scenario is that cell-ECM adhesion triggers Src kinase activation, with phosphorylated βPix then promoting the interaction of Dyn2 temporally, resulting in βPix-mediated CRC invasive migration. Interestingly, it has been reported that Dyn2 is also phosphorylated by Src and modulates its GTPase activity, endocytosis, and cancer cell invasion [57–59]. Thus, another possibility is that Src-induced Dyn2 phosphorylation may support the membrane localization of βPix-Dyn2. Additionally, the GTPase activity of Dyn2 involved in clathrin-mediated endocytosis is activated by Arf6-specific GEFs, EFA6B and EFA6D [60]. Therefore, further experiments are needed to demonstrate the impact of Dyn2 phosphorylation on βPix interaction and the role of βPix in regulating Dyn2 function, and to investigate the positive feedback mechanism of Src-βPix-Dyn2 by synergistic effects on CRC cell invasion.

For efficacious cancer treatment, numerous oncogenic proteins have been considered as molecular targets to suppress the biological functions of cancerous cells, including proliferation, differentiation and metastasis [61]. Considering that βPix functions as a tumor-promoting protein in patients with CRC, in whom βPix amplification mainly occurs among genetic mutations, we assumed that βPix could be an effective target for cancer treatment. Indeed, the delivery of anti-βPix-SH3 antibody effectively interrupted the interaction of βPix and Dyn2 and inhibited CRC invasion. Furthermore, as antibody therapy for cancer can overcome limitations of conventional chemotherapies in terms of high toxicity, weak selectivity to tumor cells and drug resistance [62], effective targeting of βPix via the intracellular delivery of anti-βPix-SH3 antibodies could afford a potential therapeutic strategy to suppress CRC progression, even in lung and breast cancers in which βPix is reportedly overexpressed [15, 63]. Further investigations assessing CRC progression using *in vivo* systems will highlight the clinical significance of the βPix-Dyn2 complex and simultaneously reinforce our findings regarding the function of the βPix-Dyn2 complex in CRC invasion using an *in vitro* human cell line model system.

In summary, our data suggest that Dyn2 serves as a novel binding partner for βPix, assisting βPix functions in CRC progression. We also verified that the spatiotemporal regulation of βPix-Dyn2 is essential for lamellipodia formation and MT1-MMP localization at the leading edge of invading cells. Thus, Dyn2-mediated spatial and Src-mediated temporal regulation of βPix activity appears to be crucial for invasive CRC migration. Furthermore, our study implies that targeting the βPix-Dyn2 complex may be an efficient strategy for the targeted treatment of cancer.

## Supplementary Information

ESM 1 (MP4 7.06 MB)

ESM 2 (MP4 10.9 MB)

ESM 3 (MP4 14.6 MB)

ESM 4 (MP4 5.95 MB)

ESM 5 (MP4 10.9 MB)

ESM 6 (PDF 1.01 MB)

## References

[1] F Bray, J Ferlay, I Soerjomataram, RL Siegel, LA Torre, A Jemal, Global cancer statistics 2018: GLOBOCAN estimates of incidence and mortality worldwide for 36 cancers in 185 countries. CA Cancer J. Clin. **68**, 394–424 (2018) 30207593 10.3322/caac.21492

[2] P.T. Caswell, T. Zech, Actin-based cell protrusion in a 3D matrix. Trends Cell Biol. **28**, 823–834 (2018) 29970282 10.1016/j.tcb.2018.06.003 PMC6158345

[3] A Nurnberg, T Kitzing, R Grosse, Nucleating actin for invasion. Nat. Rev. Cancer **11**, 177–187 (2011) 21326322 10.1038/nrc3003

[4] S Rhee, F Grinnell, P21-activated kinase 1: convergence point in PDGF- and LPA-stimulated collagen matrix contraction by human fibroblasts. J. Cell Biol. **172**, 423–432 (2006) 16449192 10.1083/jcb.200505175 PMC2063651

[5] V Sanz-Moreno, G Gadea, J Ahn, H Paterson, P Marra, S Pinner, E Sahai, CJ Marshall, Rac activation and inactivation control plasticity of tumor cell movement. Cell **135**, 510–523 (2008) 18984162 10.1016/j.cell.2008.09.043

[6] CD Lawson, AJ Ridley, Rho GTPase signaling complexes in cell migration and invasion. J. Cell Biol. **217**, 447–457 (2018) 29233866 10.1083/jcb.201612069 PMC5800797

[7] E Manser, TH Loo, CG Koh, ZS Zhao, XQ Chen, L Tan, I Tan, T Leung, L Lim, PAK kinases are directly coupled to the PIX family of nucleotide exchange factors. Mol. Cell **1**, 183–192 (1998) 9659915 10.1016/s1097-2765(00)80019-2

[8] JP ten Klooster, ZM Jaffer, J Chernoff, PL Hordijk, Targeting and activation of Rac1 are mediated by the exchange factor beta-Pix. J. Cell Biol. **172**, 759–769 (2006) 16492808 10.1083/jcb.200509096 PMC2063707

[9] T Kang, SJ Lee, Y Kwon, D Park, Loss of pix causes defects in early embryonic development, and cell spreading and platelet derived growth factor-induced chemotaxis in mouse embryonic fibroblasts. Mol. Cells **42**, 589–596 (2019) 31402636 10.14348/molcells.2019.0140 PMC6715337

[10] J. Liu, S.D. Fraser, P.W. Faloon, E.L. Rollins, J. Vom Berg, O. Starovic-Subota, A.L. Laliberte, J.N. Chen, F.C. Serluca, S.J. Childs, A betaPix Pak2a signaling pathway regulates cerebral vascular stability in zebrafish. Proc. Natl. Acad. Sci. U. S. A. **104**, 13990–13995 (2007) 17573532 10.1073/pnas.0700825104 PMC1955796

[11] T. Omelchenko, M.A. Rabadan, R. Hernandez-Martinez, J. Grego-Bessa, K.V. Anderson, A. Hall, beta-Pix directs collective migration of anterior visceral endoderm cells in the early mouse embryo. Genes Dev. **28**, 2764–2777 (2014) 25512563 10.1101/gad.251371.114 PMC4265679

[12] A Di Cesare, S Paris, C Albertinazzi, S Dariozzi, J Andersen, M Mann, R de Longhi, Curtis, p95-APP1 links membrane transport to Rac-mediated reorganization of actin. Nat. Cell Biol. **2**, 521–530 (2000) 10934473 10.1038/35019561

[13] S.R. Frank, S.H. Hansen, The PIX-GIT complex: a G protein signaling cassette in control of cell shape. Semin. Cell Dev. Biol. **19**, 234–244 (2008) 18299239 10.1016/j.semcdb.2008.01.002 PMC2394276

[14] C Plutoni, E Bazellieres, MLe Borgne-Rochet, F Comunale, A Brugues, M Seveno, D Planchon, S Thuault, N Morin, S Bodin, X Trepat, C Gauthier-Rouviere, P-cadherin promotes collective cell migration via a Cdc42-mediated increase in mechanical forces. J. Cell Biol. **212**, 199–217 (2016) 26783302 10.1083/jcb.201505105 PMC4738379

[15] SJ Ahn, KW Chung, RA Lee, IA Park, SH Lee, DE Park, DY Noh, Overexpression of betaPix-a in human breast cancer tissues. Cancer Lett. **193**, 99–107 (2003) 12691829 10.1016/s0304-3835(03)00004-1

[16] X. Lei, L. Deng, D. Liu, S. Liao, H. Dai, J. Li, J. Rong, Z. Wang, G. Huang, C. Tang, C. Xu, B. Xiao, T. Li, ARHGEF7 promotes metastasis of colorectal adenocarcinoma by regulating the motility of cancer cells. Int. J. Oncol. **53**, 1980–1996 (2018) 30132516 10.3892/ijo.2018.4535 PMC6192735

[17] A Chahdi, JP Raufman, The Cdc42/Rac nucleotide exchange factor protein beta1Pix (Pak-interacting exchange factor) modulates beta-catenin transcriptional activity in colon cancer cells: evidence for direct interaction of beta1PIX with beta-catenin. J. Biol. Chem. **288**, 34019–34029 (2013) 24129564 10.1074/jbc.M113.480103 PMC3837141

[18] H Cao, F Garcia, MA McNiven, Differential distribution of dynamin isoforms in mammalian cells. Mol. Biol. Cell **9**, 2595–2609 (1998) 9725914 10.1091/mbc.9.9.2595 PMC25532

[19] A.M. Gonzalez-Jamett, X. Baez-Matus, M.J. Olivares, F. Hinostroza, M.J. Guerra-Fernandez, J. Vasquez-Navarrete, M.T. Bui, P. Guicheney, N.B. Romero, J.A. Bevilacqua, M. Bitoun, P. Caviedes, A.M. Cardenas, Dynamin-2 mutations linked to Centronuclear Myopathy impair actin-dependent trafficking in muscle cells. Sci. Rep. **7**, 4580 (2017) 28676641 10.1038/s41598-017-04418-w PMC5496902

[20] S. Buono, J.A. Ross, H. Tasfaout, Y. Levy, C. Kretz, L. Tayefeh, J. Matson, S. Guo, P. Kessler, B.P. Monia, M. Bitoun, J. Ochala, J. Laporte, B.S. Cowling, Reducing dynamin 2 (DNM2) rescues DNM2-related dominant centronuclear myopathy. Proc. Natl. Acad. Sci. U. S. A. **115**, 11066–11071 (2018) 30291191 10.1073/pnas.1808170115 PMC6205463

[21] MY Lee, A Skoura, EJ Park, S Landskroner-Eiger, L Jozsef, AK Luciano, T Murata, S Pasula, Y Dong, M Bouaouina, DA Calderwood, SM Ferguson, P De Camilli, WC Sessa, Dynamin 2 regulation of integrin endocytosis, but not VEGF signaling, is crucial for developmental angiogenesis. Development **141**, 1465–1472 (2014) 24598168 10.1242/dev.104539 PMC3957370

[22] BS Wong, DJ Shea, P Mistriotis, S Tuntithavornwat, RA Law, JM Bieber, L Zheng, K Konstantopoulos, A direct podocalyxin-dynamin-2 interaction regulates cytoskeletal dynamics to promote migration and metastasis in pancreatic cancer cells. Cancer Res. **79**, 2878–2891 (2019) 30975647 10.1158/0008-5472.CAN-18-3369 PMC6548656

[23] C Gu, S Yaddanapudi, A Weins, T Osborn, J Reiser, M Pollak, J Hartwig, S Sever, Direct dynamin-actin interactions regulate the actin cytoskeleton. EMBO J. **29**, 3593–3606 (2010) 20935625 10.1038/emboj.2010.249 PMC2982766

[24] KM Burton, H Cao, J Chen, L Qiang, EW Krueger, KM Johnson, WR Bamlet, L Zhang, MA McNiven, GL Razidlo, Dynamin 2 interacts with alpha-actinin 4 to drive tumor cell invasion. Mol. Biol. Cell **31**, 439–451 (2020) 31967944 10.1091/mbc.E19-07-0395 PMC7185896

[25] S.M. Ferguson, P. De Camilli, Dynamin, a membrane-remodelling GTPase. Nat. Rev. Mol. Cell Biol. **13**, 75–88 (2012) 22233676 10.1038/nrm3266 PMC3519936

[26] M. Menon, O.L. Askinazi, D.A. Schafer, Dynamin2 organizes lamellipodial actin networks to orchestrate lamellar actomyosin. PLoS One **9**, e94330 (2014) 10.1371/journal.pone.0094330 PMC3978067 24710573

[27] F Chang, CA Lemmon, D Park, LH Romer, FAK potentiates Rac1 activation and localization to matrix adhesion sites: a role for betaPIX. Mol. Biol. Cell **18**, 253–264 (2007) 17093062 10.1091/mbc.E06-03-0207 PMC1751318

[28] JY Bae, SJ Ahn, JE Lee, JE Kim, MR Han, W Han, SW Kim, HJ Shin, SJ Lee, D Park, DY Noh, BetaPix-a enhances the activity of phospholipase Cgamma1 by binding SH3 domain in breast cancer. J. Cell. Biochem. **94**, 1010–1016 (2005) 15597340 10.1002/jcb.20357

[29] A. Teckchandani, J.A. Cooper, The ubiquitin-proteasome system regulates focal adhesions at the leading edge of migrating cells. Elife **5**, e17440 (2016) 10.7554/eLife.17440 PMC5092051 27656905

[30] ME Berginski, EA Vitriol, KM Hahn, SM Gomez, High-resolution quantification of focal adhesion spatiotemporal dynamics in living cells. PLoS ONE **6**, e22025 (2011) 21779367 10.1371/journal.pone.0022025 PMC3136503

[31] D. Kim, J.H. Yeom, B. Lee, K. Lee, J. Bae, S. Rhee, Inhibition of discoidin domain receptor 2-mediated lung cancer cells progression by gold nanoparticle-aptamer-assisted delivery of peptides containing transmembrane-juxtamembrane 1/2 domain. Biochem. Biophys. Res. Commun. **464**, 392–395 (2015) 26067556 10.1016/j.bbrc.2015.06.044

[32] S Kim, SH Lee, D Park, Leucine zipper-mediated homodimerization of the p21-activated kinase-interacting factor, beta Pix. Implication for a role in cytoskeletal reorganization. J. Biol. Chem. **276**, 10581–10584 (2001) 11278242 10.1074/jbc.C000806200

[33] CS Lee, CK Choi, EY Shin, MA Schwartz, EG Kim, Myosin II directly binds and inhibits Dbl family guanine nucleotide exchange factors: a possible link to Rho family GTPases. J. Cell Biol. **190**, 663–674 (2010) 20713598 10.1083/jcb.201003057 PMC2928003

[34] G.L. Razidlo, Y. Wang, J. Chen, E.W. Krueger, D.D. Billadeau, M.A. McNiven, Dynamin 2 potentiates invasive migration of pancreatic tumor cells through stabilization of the Rac1 GEF Vav1. Dev. Cell **24**, 573–585 (2013) 23537630 10.1016/j.devcel.2013.02.010 PMC3905678

[35] D. Kim, E. You, J. Jeong, P. Ko, J.W. Kim, S. Rhee, DDR2 controls the epithelial-mesenchymal-transition-related gene expression via c-Myb acetylation upon matrix stiffening. Sci. Rep. **7**, 6847 (2017) 28754957 10.1038/s41598-017-07126-7 PMC5533734

[36] J.Z. Kechagia, J. Ivaska, P. Roca-Cusachs, Integrins as biomechanical sensors of the microenvironment. Nat. Rev. Mol. Cell Biol. **20**, 457–473 (2019) 31182865 10.1038/s41580-019-0134-2

[37] Q Feng, D Baird, S Yoo, M Antonyak, RA Cerione, Phosphorylation of the cool-1/beta-Pix protein serves as a regulatory signal for the migration and invasive activity of Src-transformed cells. J. Biol. Chem. **285**, 18806–18816 (2010) 20375009 10.1074/jbc.M109.098079 PMC2881803

[38] Q Feng, D Baird, X Peng, J Wang, T Ly, JL Guan, RA Cerione, Cool-1 functions as an essential regulatory node for EGF receptor- and Src-mediated cell growth. Nat. Cell Biol. **8**, 945–956 (2006) 16892055 10.1038/ncb1453

[39] Q Feng, JG Albeck, RA Cerione, W Yang, Regulation of the Cool/Pix proteins: key binding partners of the Cdc42/Rac targets, the p21-activated kinases. J. Biol. Chem. **277**, 5644–5650 (2002) 11741931 10.1074/jbc.M107704200

[40] MHH Schmidt, K Husnjak, I Szymkiewicz, K Haglund, I Dikic, Cbl escapes Cdc42-mediated inhibition by downregulation of the adaptor molecule betaPix. Oncogene **25**, 3071–3078 (2006) 16407834 10.1038/sj.onc.1209329

[41] N. Osmani, N. Vitale, J.P. Borg, S. Etienne-Manneville, Scrib controls Cdc42 localization and activity to promote cell polarization during astrocyte migration. Curr. Biol. **16**, 2395–2405 (2006) 17081755 10.1016/j.cub.2006.10.026

[42] H Ito, T Tsunoda, M Riku, S Inaguma, A Inoko, H Murakami, H Ikeda, M Matsuda, K Kasai, Indispensable role of STIL in the regulation of cancer cell motility through the lamellipodial accumulation of ARHGEF7-PAK1 complex. Oncogene **39**, 1931–1943 (2020) 31754215 10.1038/s41388-019-1115-9

[43] N Osmani, F Peglion, P Chavrier, S Etienne-Manneville, Cdc42 localization and cell polarity depend on membrane traffic. J. Cell Biol. **191**, 1261–1269 (2010) 21173111 10.1083/jcb.201003091 PMC3010071

[44] Y Altschuler, SM Barbas, LJ Terlecky, K Tang, S Hardy, KE Mostov, SL Schmid, Redundant and distinct functions for dynamin-1 and dynamin-2 isoforms. J. Cell Biol. **143**, 1871–1881 (1998) 9864361 10.1083/jcb.143.7.1871 PMC2175237

[45] JS Herskovits, CC Burgess, RA Obar, RB Vallee, Effects of mutant rat dynamin on endocytosis. J. Cell Biol. **122**, 565–578 (1993) 8335685 10.1083/jcb.122.3.565 PMC2119668

[46] B Marks, MH Stowell, Y Vallis, IG Mills, A Gibson, CR Hopkins, HT McMahon, GTPase activity of dynamin and resulting conformation change are essential for endocytosis. Nature **410**, 231–235 (2001) 11242086 10.1038/35065645

[47] WT Chao, J Kunz, Focal adhesion disassembly requires clathrin-dependent endocytosis of integrins. FEBS Lett. **583**, 1337–1343 (2009) 19306879 10.1016/j.febslet.2009.03.037 PMC2801759

[48] G Schlunck, H Damke, WB Kiosses, N Rusk, MH Symons, CM Waterman-Storer, SL Schmid, MA Schwartz, Modulation of Rac localization and function by dynamin. Mol. Biol. Cell **15**, 256–267 (2004) 14617821 10.1091/mbc.E03-01-0019 PMC307545

[49] H Mori, T Tomari, N Koshikawa, M Kajita, Y Itoh, H Sato, H Tojo, I Yana, M Seiki, CD44 directs membrane-type 1 matrix metalloproteinase to lamellipodia by associating with its hemopexin-like domain. EMBO J. **21**, 3949–3959 (2002) 12145196 10.1093/emboj/cdf411 PMC126155

[50] Y Zhuge, J Xu, Rac1 mediates type I collagen-dependent MMP-2 activation. role in cell invasion across collagen barrier. J. Biol. Chem. **276**, 16248–16256 (2001) 11340084 10.1074/jbc.m010190200

[51] I. Masi, V. Caprara, F. Spadaro, L. Chellini, R. Sestito, A. Zancla, A. Rainer, A. Bagnato, L. Rosano, Endothelin-1 drives invadopodia and interaction with mesothelial cells through ILK. Cell Rep. **34**, 108800 (2021) 33657382 10.1016/j.celrep.2021.108800

[52] SK Donnelly, R Cabrera, SPH Mao, JR Christin, B Wu, W Guo, JJ Bravo-Cordero, JS Condeelis, JE Segall, L Hodgson, Rac3 regulates breast cancer invasion and metastasis by controlling adhesion and matrix degradation. J. Cell Biol. **216**, 4331–4349 (2017) 29061650 10.1083/jcb.201704048 PMC5716284

[53] H Hamidi, J Ivaska, Every step of the way: integrins in cancer progression and metastasis. Nat. Rev. Cancer **18**, 533–548 (2018) 30002479 10.1038/s41568-018-0038-z PMC6629548

[54] R. Mayor, S. Etienne-Manneville, The front and rear of collective cell migration. Nat. Rev. Mol. Cell Biol. **17**, 97–109 (2016) 26726037 10.1038/nrm.2015.14

[55] R.J. Eddy, M.D. Weidmann, V.P. Sharma, J.S. Condeelis, Tumor cell invadopodia: Invasive protrusions that orchestrate metastasis. Trends Cell Biol. **27**, 595–607 (2017) 28412099 10.1016/j.tcb.2017.03.003 PMC5524604

[56] EY Shin, KS Shin, CS Lee, KN Woo, SH Quan, NK Soung, YG Kim, CI Cha, SR Kim, D Park, GM Bokoch, EG Kim, Phosphorylation of p85 beta PIX, a Rac/Cdc42-specific guanine nucleotide exchange factor, via the Ras/ERK/PAK2 pathway is required for basic fibroblast growth factor-induced neurite outgrowth. J. Biol. Chem. **277**, 44417–44430 (2002) 12226077 10.1074/jbc.M203754200

[57] RD Eppinga, EW Krueger, SG Weller, L Zhang, H Cao, MA McNiven, Increased expression of the large GTPase dynamin 2 potentiates metastatic migration and invasion of pancreatic ductal carcinoma. Oncogene **31**, 1228–1241 (2012) 21841817 10.1038/onc.2011.329 PMC3218232

[58] AN Shajahan, BK Timblin, R Sandoval, C Tiruppathi, AB Malik, RD Minshall, Role of Src-induced dynamin-2 phosphorylation in caveolae-mediated endocytosis in endothelial cells. J. Biol. Chem. **279**, 20392–20400 (2004) 15007081 10.1074/jbc.M308710200

[59] H Cao, J Chen, EW Krueger, MA McNiven, SRC-mediated phosphorylation of dynamin and cortactin regulates the “constitutive” endocytosis of transferrin. Mol. Cell. Biol. **30**, 781–792 (2010) 19995918 10.1128/MCB.00330-09 PMC2812239

[60] R. Okada, Y. Yamauchi, T. Hongu, Y. Funakoshi, N. Ohbayashi, H. Hasegawa, Y. Kanaho, Activation of the small G protein Arf6 by dynamin2 through guanine nucleotide exchange factors in endocytosis. Sci. Rep. **5**, 14919 (2015) 26503427 10.1038/srep14919 PMC4621509

[61] S Piawah, AP Venook, Targeted therapy for colorectal cancer metastases: A review of current methods of molecularly targeted therapy and the use of tumor biomarkers in the treatment of metastatic colorectal cancer. Cancer **125**, 4139–4147 (2019) 31433498 10.1002/cncr.32163

[62] Y.H. Xie, Y.X. Chen, J.Y. Fang, Comprehensive review of targeted therapy for colorectal cancer. Signal Transduct. Target Ther. **5**, 22 (2020) 32296018 10.1038/s41392-020-0116-z PMC7082344

[63] N Pedersen, S Mortensen, SB Sorensen, MW Pedersen, K Rieneck, LF Bovin, HS Poulsen, Transcriptional gene expression profiling of small cell lung cancer cells. Cancer Res. **63**, 1943–1953 (2003) 12702587

