# Supplementary material for: Beta-Pix-dynamin 2 complex promotes colorectal cancer progression by facilitating membrane dynamics
Source: Cell Oncol (Dordr). 2021 Sep 28;44(6):1287–305. doi: 10.1007/s13402-021-00637-6 (PMC8648671; doi:10.1007/s13402-021-00637-6)
Supplement: Supplementary file 6 — (PDF 1.01 MB) [file 13402_2021_637_MOESM6_ESM.pdf]

Beta-Pix-dynamin 2 complex promotes colorectal cancer progression by facilitating membrane dynamics

Seula Keum[1], Soo Jung Yang[3], Esther Park[2], TaeIn Kang[2], Jee-Hye Choi[1], Jangho Jeong[1], Ye Eun Hwang[1], Jung-Woong Kim[1], Dongeun Park[2], and Sangmyung Rhee[1*]

[1]Department of Life Science, Chung-Ang University, Seoul 06974, Republic of Korea

[2]School of Life Sciences, Seoul National University, Seoul 08826, Republic of Korea

[3]Benaroya Research Institute at Virginia Mason, Translational Research Program, Seattle, WA 98101

Seula Keum and Soo Jung Yang contributed equally to this work.

*Correspondence to:

Sangmyung Rhee, Ph.D.

Department of Life Science, Chung-Ang University, Seoul 06974, Republic of Korea

Tel: +82-2-820-5818; Fax: +82-2-825-5206; E-mail: sangmyung.rhee@cau.ac.kr

Supplementary Fig. S1

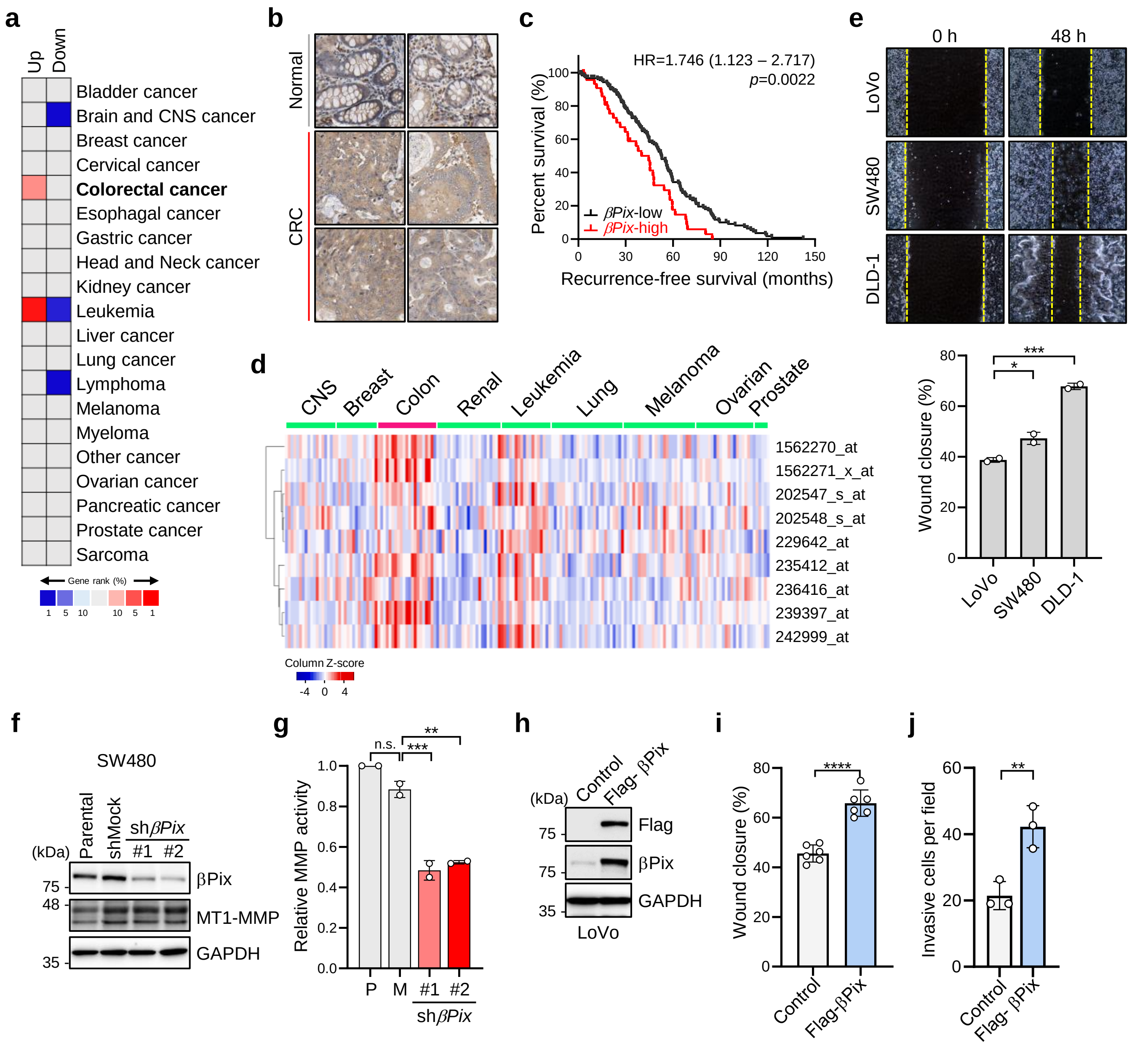

Supplementary Fig. S1 Positive correlation of βPix upregulation with CRC progression. a Profiles of βPix expression in different types of human cancer samples from Oncomine databases. Datasets were analyzed based on $p < 0.0001$, fold change >2, and gene rank = top 10%. **b** IHC analysis of βPix in normal and CRC tissues obtained from HPA databases. **c** Kaplan–Meier analysis of recurrence-free survival in patients with CRC (226 patients) showing a high expression of βPix from the GEO datasets (GSE14333). **d** Heatmap showing differentially expressed βPix in cancer cell lines from nine cancer tissue samples of different origins. GSE32474 with all probe sets of βPix was used. **e** Wound healing assay of three CRC cell lines. Wound closure was calculated by quantifying the gap area at 0 h and 48 h. Data are displayed as the mean ± standard deviation (S.D.) from two independent experiments and analyzed using one-way ANOVA with Tukey's multiple comparisons. One-way ANOVA, $F_{2,3} = 164.5$. **f** Western blotting was used to verify the knockdown of βPix in sh*βPix* #1 and #2 cell lines compared to parental and shMock, which were generated with different shRNA oligos. GAPDH was used as the loading control. **g** Secreted-MMP activity of *βPix*-knockdown cell lines measured by incubating fluorogenic MMP substrate for 2 h. Data are normalized into parental and presented as mean ± S.D. from two independent experiments. One-way ANOVA, $F_{3,4} = 133.2$. **h** Western blotting was used to verify the overexpression of Flag-βPix in LoVo cells. GAPDH was used as the loading control. **i** Wound healing assay of Flag-βPix overexpressed LoVo cells under FBS condition. The percentage of wound closure was calculated by quantifying the gap area at 0 h and 48 h. Data are displayed as the mean ± S.D. from two independent experiments and analyzed using Student's unpaired t-test. **j** The number of invasive cells from Flag-βPix overexpressed LoVo cells under FBS condition. Data are presented as the mean ± S.D. from three independent experiments. Statistical analysis was performed using Student's unpaired t-test. $^*p < 0.05$, $^{**}p < 0.01$, $^{***}p < 0.001$, $^{****}p < 0.0001$, n.s. not significant; CRC, colorectal cancer; IHC, immunohistochemistry; GEO, Gene Expression Omnibus; MMP, matrix metalloproteinase.

Supplementary Fig. S2

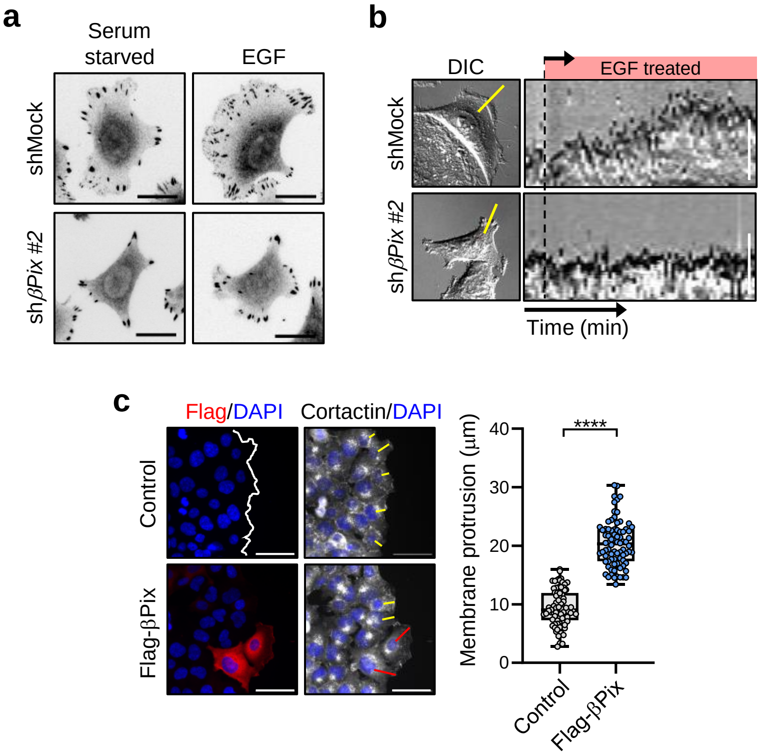

Supplementary Fig. S2 βPix-mediated membrane dynamics in CRC cell lines. a Inverted contrast images of shMock and sh*βPix* #2 SW480 cells stained for vinculin under EGF stimulation for 10 min. Scale bar, 20 μm. **b** DIC kymograph of the membrane edges in shMock and sh*βPix* #2 SW480 cells stimulated with EGF. Lined regions were used to generate the kymograph. **c** Immunofluorescence images of Flag-βPix, cortactin, and nuclei exhibit membrane protrusions at the wound edges. Scale bar, 20 μm. Yellow (in control) and red (in Flag-βPix) lines depict the length of membrane protrusions. The bar graph presents the length of membrane protrusions and the mean ± standard deviation (S.D.) from three independent experiments analyzed using Student's unpaired t-test. ****$p < 0.0001$. CRC, colorectal cancer; EGF, epidermal growth factor.

Supplementary Fig. S3

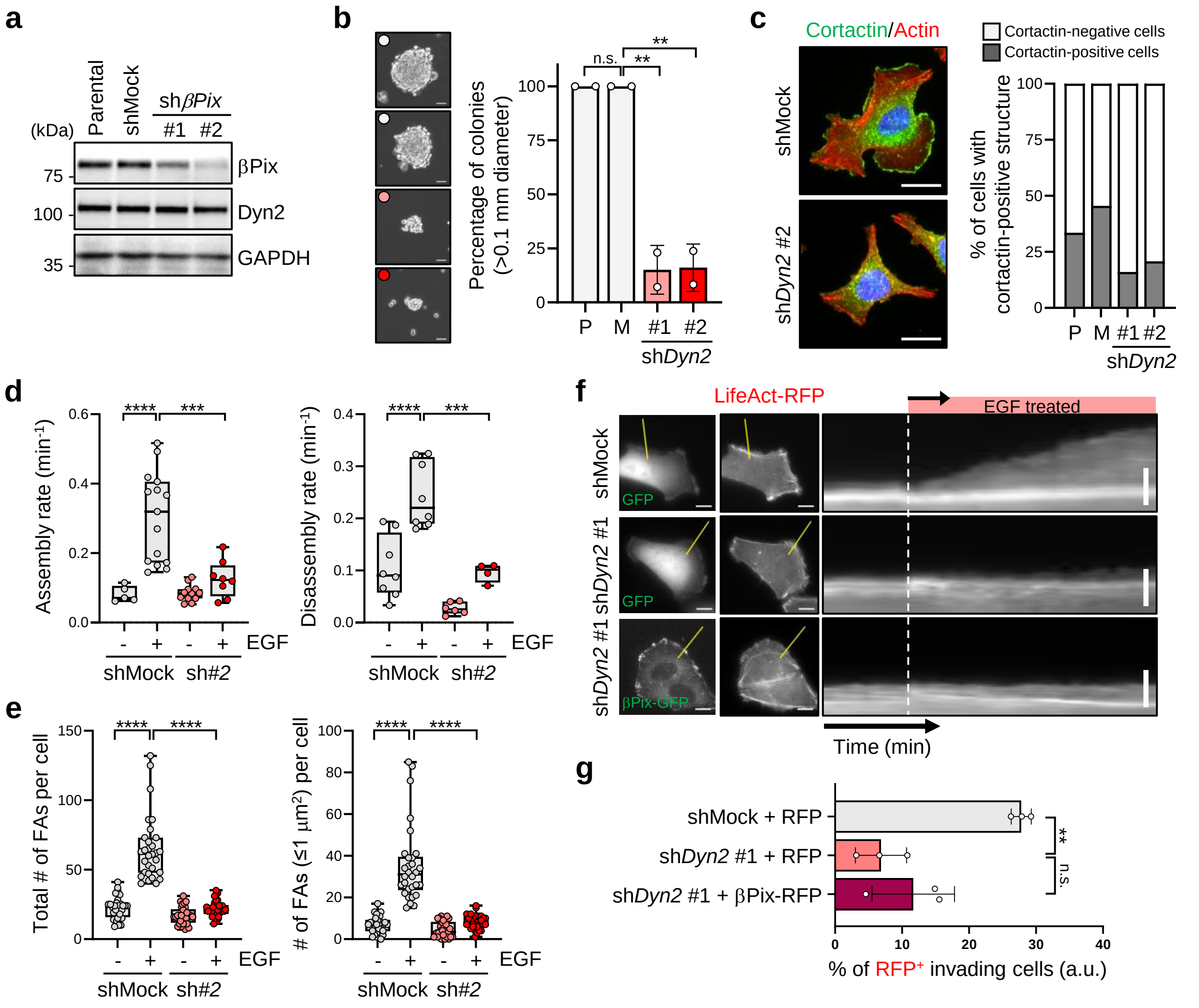

Supplementary Fig. S3 Dyn2 is required for regulation of membrane dynamics by βPix. a βPix and Dyn2 protein levels in *βPix*-silenced SW480 cells using western blotting. GAPDH was used as the loading control. **b** Phase-contrast images of *Dyn2* knockdown SW480 cell lines in the tumor sphere formation assay. The bar graph shows the percentages of tumor spheres >0.1 mm in diameter. Scale bar, 50 μm. Data are presented as the mean ± standard deviation (S.D.) from two independent experiments and analyzed using one-way ANOVA with Tukey's multiple comparisons. One-way ANOVA, $F_{3, 4} = 77.02$. **c** Immunofluorescence images of cortactin, F-actin, and nuclei in shMock and sh*Dyn2* #2 SW480 cells under EGF treatment. Scale bar, 20 μm. Bar graph depicts the percentage of cortactin-positive structures in shMock and sh*Dyn2* #2 SW480 cells. **d** FA assembly (left) and disassembly (right) rate from shMock and sh*Dyn2* #2 SW480 cells following EGF stimulation. Data are shown as the mean ± S.D. from three independent experiments. One-way ANOVA, $F_{3, 36} = 18.85$ and $F_{3, 22} = 23.92$. **e** The total number of FAs (left) and nascent FAs <1 mm^2 (right) in shMock and shDyn2 #2 SW480 cells within a 10-μm region from the leading edge following EGF treatment (n = 30, each group). One-way ANOVA, $F_{3, 116} = 89.29$ and $F_{3, 116} = 65.71$. **f** Kymography analysis of the leading edges in *Dyn2*-silenced SW480 cells expressing LifeAct-RFP with GFP or βPix-GFP following EGF stimulation. Cells were monitored for 25 min at 1-min intervals and analyzed at lined regions for kymograph. Scale bar, 10 μm (time-lapse images) and 20 μm (kymographs). **g** Transwell assay of RFP-positive invasive cells in shMock and sh*Dyn2* #1 transfected with the indicated vectors under FBS condition. The percentage of RFP$^+$ cells was calculated by dividing the number of RFP$^+$ cells in the lower chamber by total RFP$^+$ cells. Data are presented as mean ± S.D. from three independent experiments. One-way ANOVA, $F_{2, 6} = 19.77$. $^{**}p < 0.01$, $^{***}p < 0.001$, $^{****}p < 0.0001$, n.s. not significant; EGF, epidermal growth factor; FA, focal adhesion.

Supplementary Fig. S4

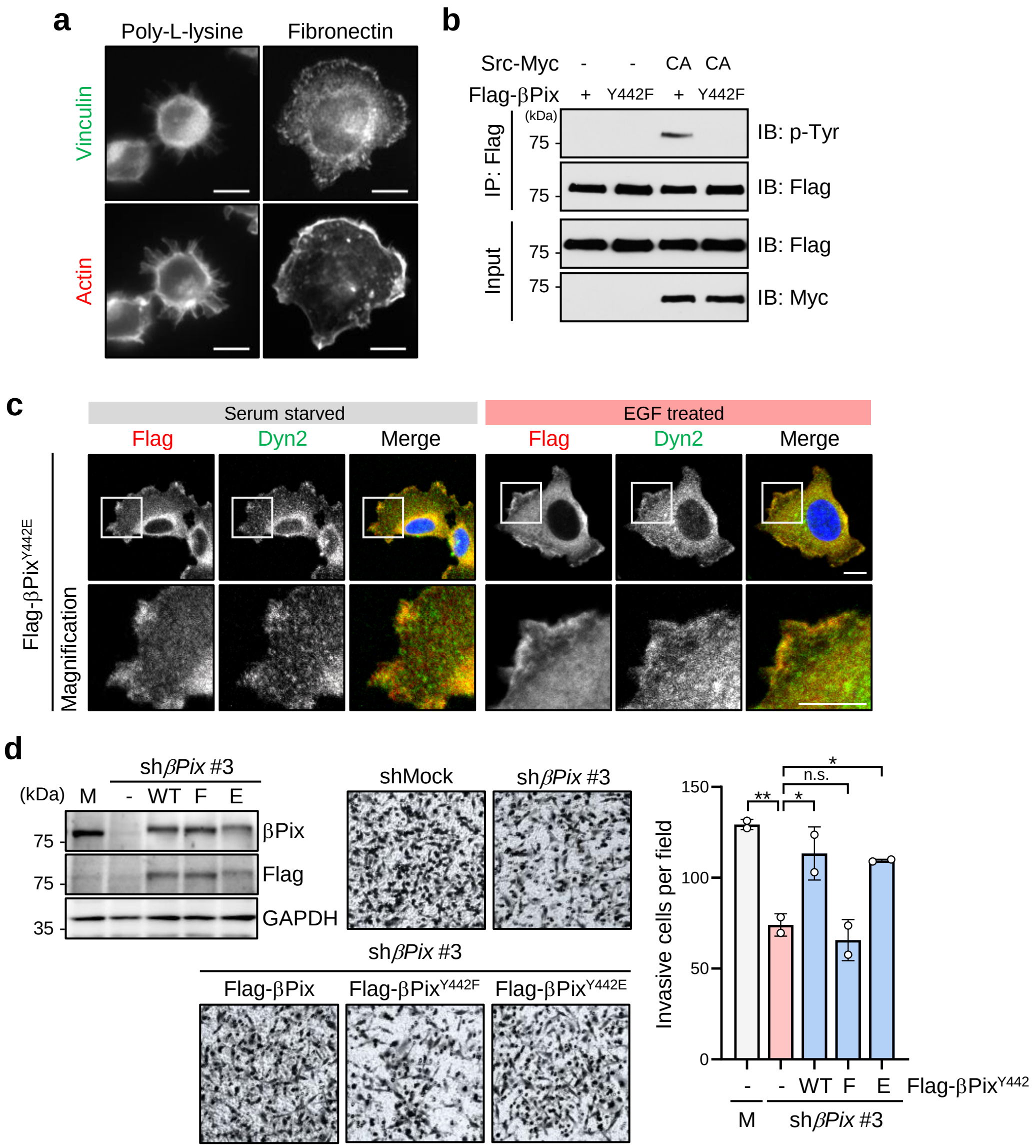

Supplementary Fig. S4 Increased membrane localization of βPix-Dyn2 complex and cell invasion on restoring βPix Y442 phosphorylation. a Immunofluorescence images of vinculin and F-actin in HEK293T cells incubated on poly L-lysine and fibronectin-coated dishes for 15 min after 1 h in suspension. Scale bar, 20 μm. **b** Western blotting was used to verify βPix Y442 phosphorylation by constitutively active Src using Flag-βPix WT and Y442F mutant. HEK293T cells were transfected with indicated vectors and immunoprecipitated with anti-Flag antibodies, followed by immunoblotting of Flag and phospho-tyrosine (p-Tyr). **c** Immunofluorescence images of endogenous Dyn2 and Flag-βPix Y442E in SW480 cells under EGF treatment for 10 min. Magnification indicates the enlarged images from the boxed regions. Scale bar, 20 μm (in all images). **d** Representative images in Transwell assay of *βPix*-knockdown SW480 cells with overexpression of Flag-βPix WT, Y442F, or Y442E under FBS condition. Western blotting in the left panel shows overexpressed βPix. The number of invasive cells was calculated by counting the cells at the bottom of the chambers, and the data were analyzed using one-way ANOVA with Tukey's multiple comparisons. The results are presented as the mean ± standard deviation (S.D.) from two independent experiments. One-way ANOVA, $F_{4, 5}$ = 19.16. *p < 0.05, **p < 0.01, n.s. not significant; EGF, epidermal growth factor.

Supplementary Fig. S5

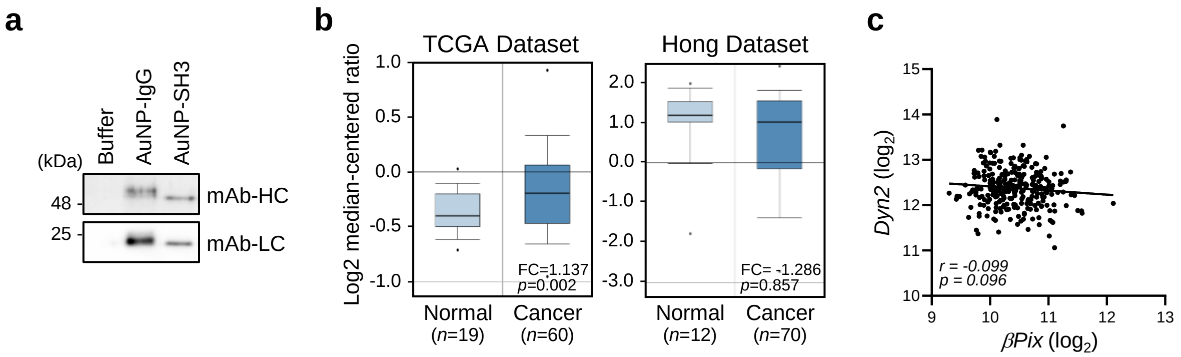

Supplementary Fig. S5 Analysis of the correlation between βPix and Dyn2 in patients with CRC. a Western blotting to examine the conjugation of IgG and anti-βPix SH3 antibodies with AuNPs. Conjugated antibodies at AuNP were visualized with HRP-bound secondary antibodies by detecting heavy and light chains of antibodies. **b** Box plots of Dyn2 expression in CRC tissues compared with normal tissues from Oncomine databases. **c** Correlation plot between βPix and Dyn2 obtained from TCGA datasets. The expression of genes is shown as a $\log_2$ value. *r* indicates Pearson's correlation coefficient, and the *p*-value is calculated from *r*. CRC, colorectal cancer; mAb, monoclonal mouse antibodies; HC, heavy chain of antibody; LC, light chain of antibody; TCGA, The Cancer Genome Atlas.

Supplementary Table S1. Off-target efficiency of shRNA oligos in SW480 cells

shRNA	Number of off-targets	Gene name	Identity† (%)	Fold change‡ (Mean ± S.D.)	*p*-value§
sh*βPix* #1	Off-target #1	*PTCH2*	73	1.385 ± 0.142	0.06203
	Off-target #2	*TRADD*	68	1.098 ± 0.034	0.05507
	Off-target #3	*CPSF3*	63	1.040 ± 0.043	0.62071
sh*βPix* #2	Off-target #1	*AKAP6*	84	1.013 ± 0.043	0.72165
	Off-target #2	*FBXW2*	78	0.687 ± 0.103	0.04954
	Off-target #3	*SUV39H2*	73	1.037 ± 0.034	0.26309
sh*βPix* #3	Off-target #1	*CREG2*	85	ND¶	-
	Off-target #2	*NBAS*	66	1.192 ± 0.091	0.09719
	Off-target #3	*LIN7A*	61	ND	-
sh*Dyn2* #1	Off-target #1	*PPIL2*	71	1.245 ± 0.013	0.00005
	Off-target #2	*TBXAS1*	66	ND	-
	Off-target #3	*HOPX*	66	ND	-
sh*Dyn2* #2	Off-target #1	*USP28*	66	1.135 ± 0.375	0.66109
	Off-target #2	*ANKFN1*	66	ND	-
	Off-target #3	*MBD2*	66	0.602 ± 0.1	0.02990

† The percent identity describes the similarity of off-target genes to shRNA sequence.
‡ Fold change represents the ratio of off-target genes in knockdown cells compared to shMock cells. Fold change shows the mean ± S.D. from two independent experiments.
§ p-value was calculated by Student's unpaired t-test.
¶ ND: not detected

Supplementary Table S2. The list of primers used for qPCR

Gene name		Primer sequence (5' - 3')
PTCH2	F	ATGACAGTGGAACTCTTTGGT
	R	ACTGTGAACTCAACGCCAA
TRADD	F	GAAATCTGAAGTGCGGCTC
	R	TGACCCTGGAACAGAAAAGT
CPSF3	F	CGTTTACAGCAAGAGGTTGG
	R	TCCAAGTTAAGGTTGGCAGT
AKAP6	F	CAATGCCACTACAAGCAACA
	R	TCCTGAGAGGAAGGACTTGA
FBXW2	F	TGGCCAATTGGGAGAGAAAT
	R	GGTAGAGACCAAGTGCTGAA
SUV39H2	F	AGCTGTGACCCAAATCTTCA
	R	TCAGCTCTTCTCCAGCATTT
CREG2	F	GGTGGCTGATCTGATGAAGA
	R	TGAGCGTTAACTGGACACAT
NBAS	F	TGAAGAGAACCGCTACTGTC
	R	CTTTTCATAGGTGGCCAAGC
LIN7A	F	CTGCTATCAGTGAACGGAGT
	R	GAACTTTTGGGGTGTATCGC
PPIL2	F	CCTACCTGGACAAGAAGCAT
	R	TCAGTTTTGGGGTCACTCTC
TBXAS1	F	CAGCTTTCAGATTCACACGG
	R	CCTTTCAGGGTTGAAGGTCT
HOPX	F	GGTGGAAATCCTGGAGTACA
	R	GGGTCTCCTCCTCGGAAA
USP28	F	TCCCCTGCATTCACCTTATC
	R	GTAGAAACTCCCCTAGGCAC
ANKFN1	F	TAGACTGTCTTCCATCCCCA
	R	TGGAGTCGTAGTCACTGTTG
MBD2	F	TCAGACCCACAACGAATGAA
	R	CTCCTTGAAGACCTTTGGGT
